# Enhancing bacterial fitness and recombinant enzyme yield by engineering the quality control protease HtrA of *Bacillus subtilis*

**DOI:** 10.1128/spectrum.01778-23

**Published:** 2023-10-11

**Authors:** Ayşegül Öktem, David Núñez-Nepomuceno, Borja Ferrero-Bordera, Jonathan Walgraeve, Michael Seefried, Manuela Gesell Salazar, Leif Steil, Stephan Michalik, Sandra Maaß, Dörte Becher, Ulrike Mäder, Uwe Völker, Jan Maarten van Dijl

**Affiliations:** 1 Department of Medical Microbiology, University of Groningen, University Medical Center Groningen, Groningen, The Netherlands; 2 Interfaculty Institute for Genetics and Functional Genomics, University Medicine Greifswald, Greifswald, Germany; 3 Department of Microbial Proteomics, University of Greifswald, Greifswald, Germany; 4 Institute of Microbiology, University of Greifswald, Greifswald, Germany; 5 Molecular Biology Department, AB Enzymes, Darmstadt, Germany; Forschungszentrum Jülich GmbH, Juelich, Germany

**Keywords:** *Bacillus subtilis*, secretion stress, HtrA, alpha amylase

## Abstract

*Bacillus subtilis* is a Gram-positive bacterium that is well-known for its high secretory capacity. Although secretion of homologous proteins is extremely efficient, secretion of heterologous proteins imposes what is known as “secretion stress” on the cells. This is not desirable in an industrial setting, because the bacterial secretion stress responses can adversely affect product yields. So far, the main approach to reduce such counterproductive secretion stress responses has been to remove components of the CssR-CssS two-component regulatory system, including the CssR-CssS-regulated quality control proteases HtrA and/or HtrB. However, there are several examples indicating that a complete loss of these proteases does not correlate with improved protein secretion or a reduced secretion stress response. In this study, an alternative approach to limit the effects of secretion stress by modulating the protease activity of HtrA rather than completely removing this protease was investigated. Indeed, expression of the proteolytically inactive HtrA led to a generally improved fitness of the bacteria when producing the heterologous α-amylase AmyQ. Moreover, compared to control strains, the strain expressing the mutant HtrA provided higher AmyQ yields, which correlated with superior α-amylase enzymatic activity in shake flasks and fermenter-scale cultivations. Proteome analysis of the engineered strains revealed critical differences in stress responses and metabolism. Altogether, our present findings show how proteolytically inactive HtrA can be employed to achieve improved bacterial fitness and higher enzyme yields.

**IMPORTANCE:**

In the expanding market of recombinant proteins, microbial cell factories such as *Bacillus subtilis* are key players. Microbial cell factories experience secretion stress during high-level production of secreted proteins, which can negatively impact product yield and cell viability. The CssRS two-component system and CssRS-regulated quality control proteases HtrA and HtrB play critical roles in the secretion stress response. HtrA has a presumptive dual function in protein quality control by exerting both chaperone-like and protease activities. However, its potential role as a chaperone has not been explored in *B. subtilis*. Here, we describe for the first time the beneficial effects of proteolytically inactive HtrA on α-amylase yields and overall bacterial fitness.

## INTRODUCTION

The global market size for recombinant proteins in 2021 was 1.75 billion USD, and it is expected to reach 3.93 billion USD by the year 2030 (https://www.globenewswire.com/en/news-release/2022/06/03/2456023/0/en/Recombinant-Protein-Market-worth-3-93-Billion-by-2030-Registering-a-CAGR-of-10-12.html). Microbial cell factories are predicted to become even more prevalent in this expanding market (1). However, protein quality control and scale-up are two outstanding challenges in industrial recombinant protein production. The quality of the end product is critical and depends on the efficiency of protein folding. Heterologous or engineered proteins may fold slowly or form misfolded aggregates, becoming targets of extracellular proteases. Importantly, chaperones can reduce the folding time and aid in correct folding. This will protect proteins from degradation, resulting in increased product yield and quality. Although scale-up is necessary for large-scale production of the recombinant proteins, it can affect protein production and quality control. Therefore, extensive optimization is required for each strain to be able to perform well in a large-scale fermentation setting. Still, it is a common problem that strains performing well in small-scale experiments do not show comparable performance upon scale-up. There are many reasons for this phenomenon including enhanced stress exposure, metabolic shifts, and issues with genetic stability (2). Especially in large-scale fermentation, the cells are exposed to a more heterogeneous substrate availability, when compared to small-scale fermentation. Additionally, in large-scale fermentation, compartments with differential pressure impact the oxygen availability. Therefore, it is crucial to test the strain performance at different levels of scale-up.

Secretion stress is one of the main stresses encountered by microbial cell factories during high-level production of secreted proteins, and it specifically refers to the stress caused by accumulation of unfolded (heterologous) proteins (3). In the Gram-positive bacterial cell factory *Bacillus subtilis,* the CssR-CssS two-component system (TCS) is responsible for mounting an adequate secretion stress response that involves expression of the quality control proteases HtrA and HtrB (4, 5). CssS is a sensor histidine kinase that detects misfolded and aggregated proteins at the membrane-cell wall interface. Subsequently, CssS phosphorylates CssR, thereby activating it. Phosphorylated CssR acts as a transcription regulator to enhance expression of the *htrA* and *htrB* genes (4). A range of heterologous proteins, including the α-amylase AmyQ of *Bacillus amyloliquefaciens*, as well as heat shock, are known to activate the CssR-CssS TCS (5 – 7). Deletion of the *cssRS*, *htrA*, and *htrB* genes has been investigated in terms of recombinant protein yields, bacterial stress levels, and bacterial temperature sensitivity. However, these findings suggest that manipulating the CssR-CssS system by deleting the respective components does not improve secretory protein production (4, 8).

The HtrA family of proteases is found in all domains of life (9). HtrA homologs in prokaryotes prevent the accumulation of unfolded proteins (5, 10). In *B. subtilis*, HtrA and its cross-regulated paralog HtrB counteract secretion stress (4, 5, 7). In bacterial pathogens, HtrA-like proteases are crucial for survival in host organisms, which makes them interesting targets for treatment (11 – 13). In humans, HtrA family proteases are implicated in pathological conditions, such as cancer and Parkinson’s or Alzheimer’s disease (14). Effects of amino acid substitutions that impair HtrA’s protease activity have been tested in various organisms. Such mutants are sensitive to high temperatures as shown for *Escherichia coli* (15). In pathogens, such as *Helicobacter pylori*, mutation of the protease domain of HtrA affects survival under stress conditions (16). On the other hand, proteolytically inactive HtrA was shown to have potent chaperone-like activity in *E. coli* (17, 18). Interestingly, in *B. subtilis*, the inactivation of HtrA leads to increased thermotolerance (7). This suggests that a chaperone-like activity of the inactive HtrA can combat the detrimental effects of heat stress, which is generally considered to represent a form of protein unfolding stress (19, 20). On the other hand, the loss of HtrA causes induction of HtrB (4, 21), which may lead to enhanced degradation of the not yet tightly folded secretory proteins when they emerge from the Sec translocation channel.

Until now, the potentially beneficial effects of proteolytically inactive HtrA on secretion stress management and recombinant protein production have not been investigated. Therefore, the present study was aimed at exploring the impact of proteolytically inactive HtrA on protein production using the major industrial cell factory *B. subtilis* as a model system. In brief, we show that proteolytically inactive HtrA enhances bacterial fitness and recombinant enzyme production, in particular upon scale-up.

## RESULTS AND DISCUSSION

### Real-time monitoring of secretion stress during microtiter plate cultivation

HtrA is a serine protease with a conserved catalytic domain, where the Ser^260^ residue serves as the nucleophile responsible for substrate hydrolysis (22, 23). Accordingly, a proteolytically inactive HtrA derivative was obtained by replacing the Ser^260^ residue with Ala. To achieve constitutive expression of the mutated HtrA (designated H^m^) or wild-type (WT) HtrA (designated H^WT^), the respective genes were cloned in plasmid pHB201 and placed under control of the p59 promoter. The resulting plasmids were introduced in a chromosomal ∆*htrA* background. To study the secretion stress responses in real time, a luciferase-based reporter system was built, using the *htrA* promoter (P*htrA*) from *B. subtilis* that is controlled by the CssRS TCS. Accordingly, this promoter can be used to monitor secretion stress (7). To this end, P*htrA* was cloned upstream of the *luxABCDE* operon from *Photorhabdus luminescens* on the integrative plasmid pBS3Clux (24). The reporter strain was then used to investigate the effects of secretion stress imposed through production of the heterologous α-amylase AmyQ from plasmid pKTH10 and constitutive expression of WT or mutant HtrA from plasmid pHB201.

**Fig 1:**
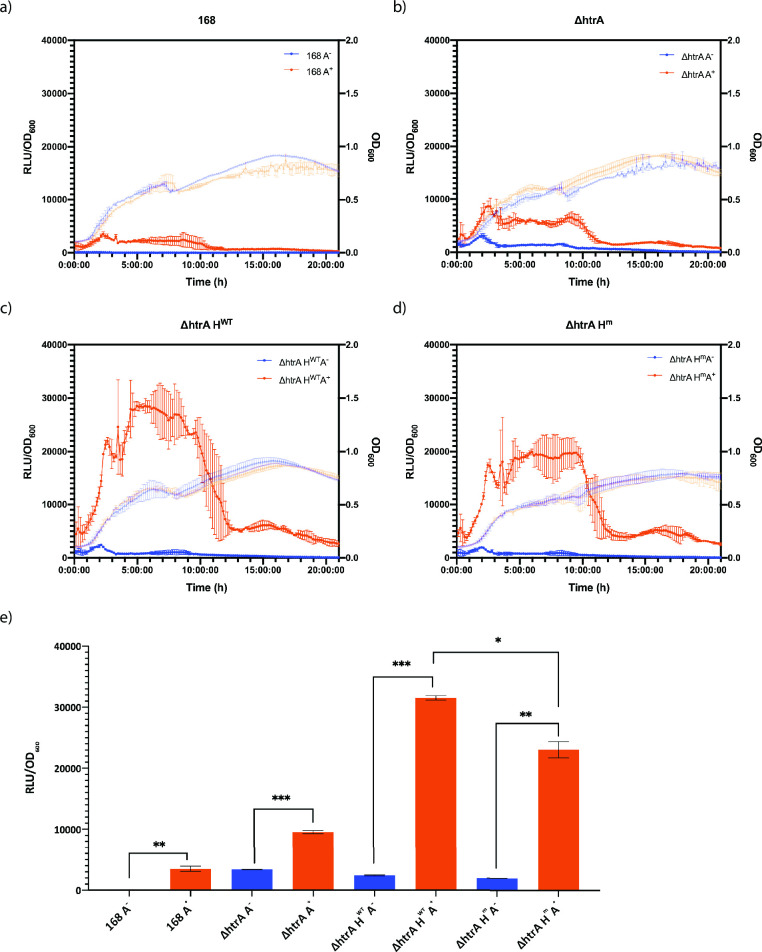
*HtrA* promoter activity in *B. subtilis*. Exponentially growing cells of the *B. subtilis* strains 168, Δ*htrA*, Δ*htrA* H^WT^, and Δ*htrA* H^m^ with (A^+^) and without (A^−^) the plasmid pKTH10 were diluted to OD_600_ of 0.05 in LB, and 150-µL aliquots were transferred to a black 96-well plate with clear bottom (Thermo Scientific). The luminescence and OD_600_ were measured every 10 min for 24 h in a BioTek Synergy 2 plate reader (37°C, continuous shaking). (a to d) Real-time measurements of relative light intensity units (RLU)/OD_600_ are plotted on the left *y*-axis, with data points represented by circles. OD_600_ measurements are plotted on the right *y*-axis, with data points represented by triangles. (e) Maximum RLU/OD_600_ values of the strains. Blue bars represent strains without the plasmid pKTH10 (A^−^), and orange bars represent strains with the plasmid pKTH10 (A^+^). Data points and error bars indicating the mean values and SEM of three independent experiments are presented. Student’s *t*-test was used to determine significant changes (*0.01 < *P* < 0.05; **0.001 < *P* < 0.01; ***P < 0.001).

Secretion stress induced by AmyQ production (designated A^+^) increased during exponential growth in all strains and reached a maximum for the 168 A^+^ and Δ*htrA* A^+^ strains during the transition to stationary phase (Fig. 1a and b). Notably, secretion stress induction could be observed in the ∆*htrA* strain without amylase production (designated A^−^; Fig. 1b), which is in line with the notion that the absence of HtrA is a trigger for induction of the *htrA* promoter (4). The secretion stress peaked at transition phase with a second peak during the early stationary phase in the Δ*htrA* H^WT^A^+^ and Δ*htrA* H^m^A^+^ strains (Fig. 1c and d).

The engineered Δ*htrA* H^WT^A^+^ and Δ*htrA* H^m^A^+^ strains showed a notably higher *htrA* promoter activity than the 168 A^+^ and Δ*htrA* A^+^ strains (Fig. 1e, orange bars). This effect was not due to the expression of HtrA from the plasmid, since strains containing only the HtrA expression vector (Δ*htrA* H^WT^A^−^ and Δ*htrA* H^m^A^−^) and the empty ∆*htrA* strain (Δ*htrA* A^−^) presented comparable *htrA* promoter activity (Fig. 1e, blue bars). Therefore, the high-level *htrA* promoter activity during AmyQ production in the Δ*htrA* H^WT^A^+^ and Δ*htrA* H^m^A^+^ strains is most likely due to constitutive expression of HtrA^WT^ or HtrA^m^, instead of the genomic inducible system in the WT strain. Interestingly, the Δ*htrA* H^m^A^+^ strain displayed significantly lower *htrA* promoter activity than the Δ*htrA* H^WT^A^+^ strain (Fig. 1e). This shows that, under the same conditions, the strain expressing the proteolytically inactive HtrA experienced less secretion stress.

### Amylase yield and cellular stress during shake flask cultivation

To investigate how the proteolytically inactive HtrA impacts secretion stress and amylase production, shake flask experiments were performed using a Δ*htrA* background strain. In this case, the SigE-regulated *yqfD* gene, which is essential for sporulation (25), was also deleted as a step-up toward subsequent fermenter experiments that mimic industrial conditions, where sporulation-deficient strains are the norm (26). For the shake flask cultivations, 1-L baffled shake flasks were used, and samples were withdrawn after 3, 6, and 18 h from the start of the main culture. These time points corresponded to the exponential, transition, and late stationary growth phases, respectively. The strains included in this experiment were (i) a strain containing the two empty plasmids to represent the condition where HtrA and AmyQ are both absent (H^−^A^−^); (ii) a strain lacking HtrA but producing AmyQ (H^−^A^+^); (iii) a strain producing both WT HtrA and AmyQ (H^WT^A^+^); and (iv) a strain producing both the proteolytically inactive HtrA and AmyQ (H^m^A^+^). Selective antibiotics were included in the lysogeny broth (LB) medium to prevent the loss of plasmids carrying the *htrA* or *amyQ* genes.

**Fig 2:**
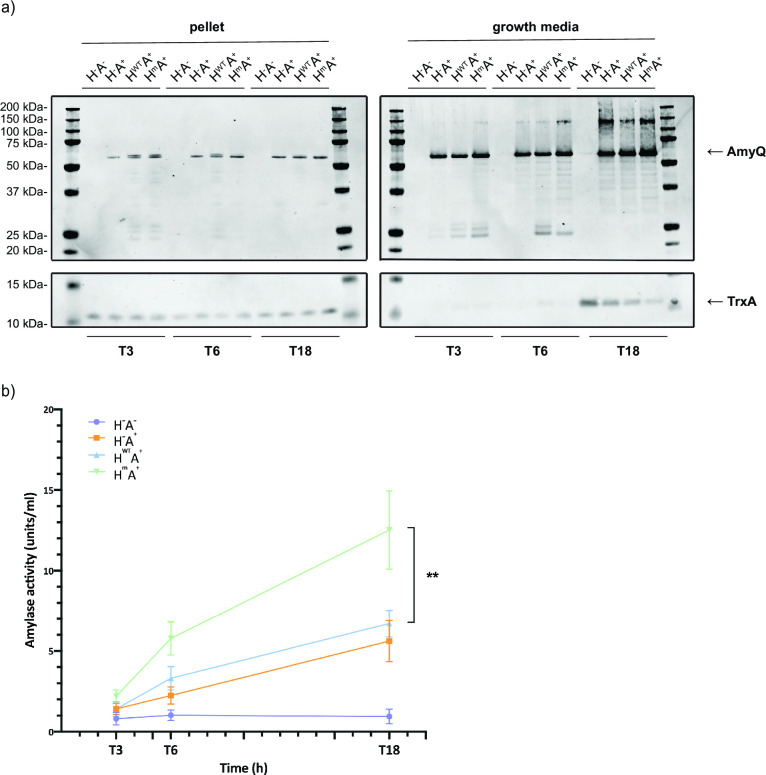
AmyQ production and activity in shake flask cultures of *B. subtilis. B. subtilis* strains H^−^A^−^, H^−^A^+^, H^WT^A^+^, and H^m^A^+^ were grown overnight in LB medium supplemented with antibiotics (37°C, 250 rpm). Pre-cultures were started from overnight cultures by diluting 10-fold in LB supplemented with antibiotics. When a pre-culture reached the exponential growth phase, the main culture was started by standardizing the cultures to an OD_600_ of 0.1 in LB supplemented with antibiotics. Samples were withdrawn after 3, 6, and 18 h from the start of the main culture. (a) Samples were standardized at OD_600_ of 2, and cells were separated from the growth medium by centrifugation. The supernatant fraction was used for trichloroacetic acid precipitation, and cell pellets were disrupted by bead beating. Proteins from the cell pellet and growth medium fractions were separated by LDS-PAGE. AmyQ and TrxA levels were assessed by Western blotting with AmyQ- and TrxA-specific polyclonal antibodies, respectively. The cytoplasmic TrxA protein was used as a loading control for cell pellet samples and as a cell lysis marker for growth medium samples. (b) α-Amylase activity in the growth medium fraction of standardized samples was measured by following the amylase activity assay kit instructions (α-Amylase Assay Kit, Megazyme Cat. No. K-CERA).

First, the amylase production and the secreted amylase enzymatic activity were assessed. AmyQ protein levels in the cells and the growth media were detected by Western blotting using a polyclonal AmyQ-specific antibody. The H^m^A^+^ strain showed increased levels of the secreted AmyQ protein at all time points during growth compared to the H^−^A^+^ and H^WT^A^+^ strains (Fig. 2a, right panel). Here, it is noteworthy that we observed differences in the AmyQ degradation patterns in the growth medium fractions, suggesting differences in protein quality control between the investigated strains (Fig. 2a, right panel). To gain insight how HtrA^m^ influences the activity of the secreted AmyQ, an α-amylase activity assay was employed. Importantly, the H^m^A^+^ strain showed twofold higher amylase activity than the H^WT^A^+^ and H^−^A^+^ strains (Fig. 2b). These observations show that production of the proteolytically inactive HtrA has a significantly positive impact on the production level and quality of the secreted AmyQ, resulting in a higher yield of enzymatically active AmyQ.

**Fig 3:**
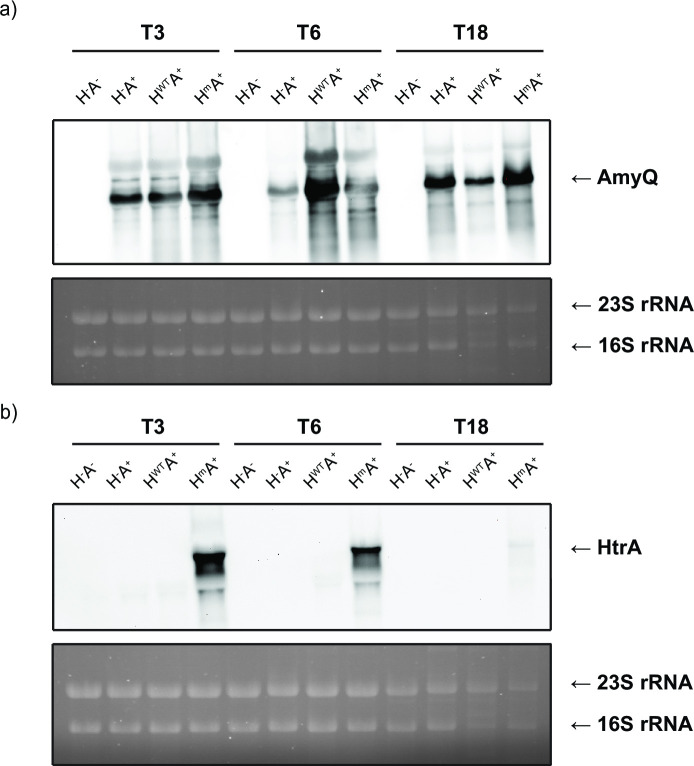
AmyQ and HtrA mRNA levels in shake flask cultures *of B. subtilis. B. subtilis* strains H^−^A^−^, H^−^A^+^, and H^WT^A^+^, H^m^A^+^ were grown overnight in LB medium supplemented with antibiotics (37°C, 250 rpm). Pre-cultures were started from the overnight cultures by diluting 10-fold in LB supplemented with antibiotics. When the pre-culture reached the exponential growth phase, the main culture was started by standardizing the cultures to an OD_600_ of 0.1 in LB supplemented with antibiotics. Samples were withdrawn after 3, 6, and 18 h from the start of the main culture. Sixteen OD units of bacterial culture were harvested, and RNA extraction was carried out. Four micrograms of total RNA was loaded in each well. (a) *amyQ* and (b) *htrA* transcripts were detected with digoxygenin-labeled antisense RNA probes. In the bottom panels, the rRNA is visualized as a control for gel loading.

Next, to determine the *amyQ* and *htrA* mRNA levels by Northern blotting, RNA was extracted from samples that were collected at the same time points from the same cultures used in the afore-described Western blotting experiments. Transcripts of *amyQ* were detected in all strains containing the pKTH10 plasmid (A^+^) (Fig. 3a). Interestingly, the H^WT^A^+^ strain showed the highest *amyQ* transcript level at T6, but this clearly did not translate into the highest AmyQ protein levels or activity (Fig. 2b and 3a). The H^m^A^+^ strain showed the highest *amyQ* transcript levels at T3 and T18 (Fig. 3a). Surprisingly, *htrA* transcripts were not detectable in the H^WT^A^+^ strain, even though this strain was grown under antibiotic pressure for plasmid maintenance, as was the case for the other strains (Fig. 3b). This was suggestive of a strong counterselection against constitutive expression of both the HtrA^WT^ and AmyQ, whereas such a counterselection would be absent from the H^m^A^+^ strain producing both the mutant HtrA and AmyQ (Fig. 3a and b).

To investigate if there could be counterselection against constitutive co-expression of HtrA^WT^ and AmyQ, a shake flask cultivation experiment was performed in the absence of selective antibiotic pressure. Indeed, as shown by plating on selective media, the H^WT^A^+^ strain lost the pKTH10 plasmid completely within 5 h, whereas the H^m^A^+^ strain stably maintained both plasmids (Fig. S1a and b). The rapid loss of the high-copy pKTH10 plasmid is in line with previous investigations on plasmid stability, which had shown that pUB110-derived plasmids such as pKTH10 suffer from segregational instability in *B. subtilis* (27). To understand why no *htrA* mRNA was detectable in the H^WT^A^+^ strain cultured in the presence of selective antibiotics for the plasmids encoding *htrA* or *amyQ*, we plated this strain and the H^m^A^+^ strain upon shake flask cultivation and subsequently picked colonies for whole-genome sequencing. The sequencing results revealed large deletions in the plasmid pHB201_WT htrA covering the promoter region and most of the WT *htrA* gene, whereas such deletions were not observed for the plasmid carrying the mutated *htrA* gene (Fig. S1c). Together, these observations demonstrate that there is indeed a strong counterselection against the constitutive co-expression of the WT HtrA and AmyQ.

**Fig 4:**
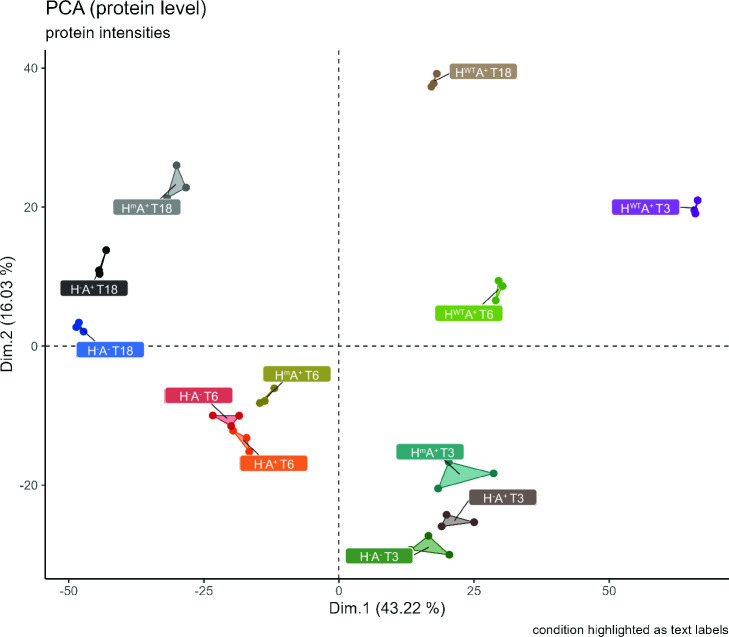
Principal component analysis (PCA) of the LFQ protein intensities of *B. subtilis* grown in shake flasks. Three biological replicates of each condition are marked with the same color.

Lastly, we wanted to decipher the underlying physiological changes that led to the differential protein quality control in the H^−^A^−^, H^−^A^+^, H^WT^A^+^, and H^m^A^+^ strains. To this end, we performed a proteome profiling of the different cells collected during the shake flask cultivations. All samples were collected in biological triplicates. According to a principal component analysis (PCA) of the obtained mass spectrometry (MS) data, the biological replicates of each strain clustered together more closely than the replicates of different strains. In particular, clusters of protein patterns of these strains were separated by sampling time, explained by the first dimension (43.22%), and by strain, as explained by the second dimension (16.03%) (Fig. 4). This observation showed that there are clear differences in the protein composition of the different strains. However, it should be noted that, in the PCA, the H^WT^A^+^ strain was grouped completely separately from the other three strains (H^–^A^–^, H^–^A^+^, and H^m^A^+^) indicating major differences in protein composition (Fig. 4).

**Fig 5:**
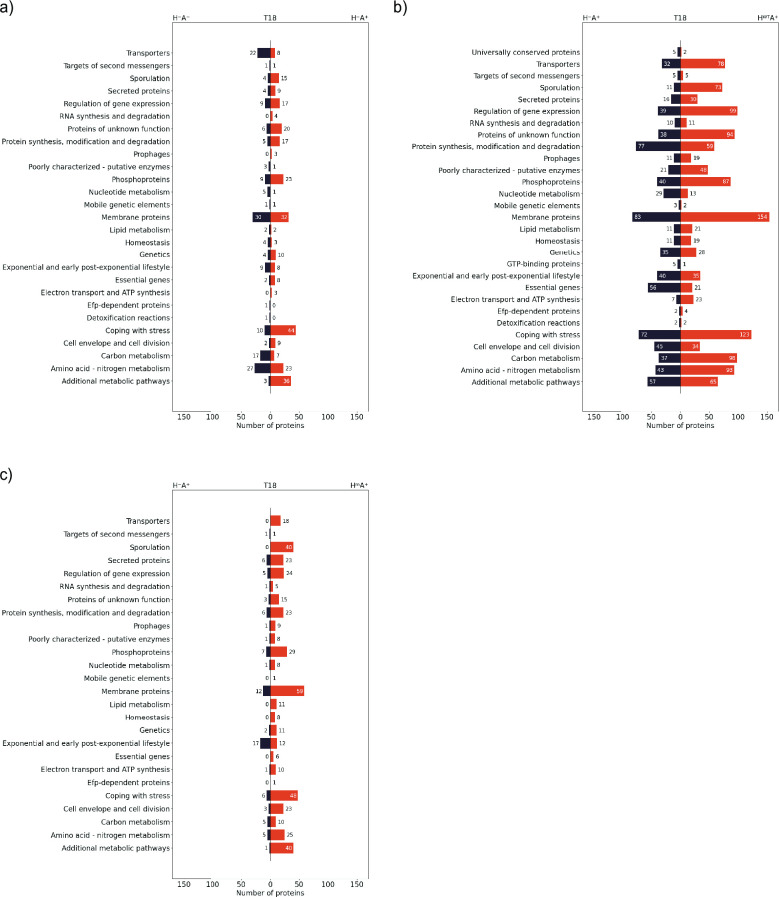
Numbers of *B. subtilis* proteins belonging to particular functional categories and presenting significantly different abundances in T18 samples. Proteins with *P* values smaller than 0.05 and fold changes greater than 1.5 were plotted according to the SubtiWiki functional categories they belong to. Comparison of (a) H^−^A^−^ and H^−^A^+^, (b) H^−^A^+^ and H^WT^A^+^, and (c) H^−^A^+^ and H^m^A^+^ strains at T18 is shown. The numbers of proteins in each category are shown on the bars.

At the proteome level, we first evaluated the impact of AmyQ production in the Δ*htrA* background (H^−^). During the exponential growth phase (T3), the most striking effect observed for the H^−^A^−^ strain was the higher abundance of CodY- and TnrA-regulated proteins, which are involved in amino acid and nitrogen metabolism, compared to the H^−^A^+^ strain (Fig. S2a). These proteins included IlvB, IlvC, IlvH, LeuB, LeuC, and LeuD, which were 3.5-, 2.9-, 3.7-, 2.8-, 3.2-, and 3.3-fold more abundant in H^−^A^−^ strain than H^−^A^+^ strain, respectively (Table S1). The strongest effects of AmyQ production were visible during the late stationary phase (T18; Fig. 5a; Fig. S3). At T18, proteins related to coping with stress and additional metabolic pathways were more abundant in the H^−^A^+^ strain when compared to H^−^A^−^ strain, while strong differential effects were observed for membrane proteins and amino acid and nitrogen metabolism (Fig. 5a). As expected, proteins involved in the typical AmyQ-induced secretion stress response, such as CssS, CssR, and HtrB, were 1.5-, 1.6-, and 1.6-fold more abundant in the H^−^A^+^ strain than in the H^−^A^−^ strain, respectively (Fig. S2b and Table S1). When the identified proteins were grouped according to regulons, a majority of the proteins repressed by AhrC, CcpA, DegU, and IolR, or activated by AbrB and Spx, were more abundant in the H^−^A^+^ strain compared to the H^−^A^−^ strain at T18 (Fig. S2c). Additionally, YlxR-regulated proteins such as ArtP, R, and Q; ArgB, C, D, F, G, H, and J; and HisA, B, D, F, G, I, and Z were impacted by the production of AmyQ since they were more abundant in the H^−^A^+^ strain compared to H^−^A^−^ strain (Table S1).

Consistent with the results from the PCA, massive differences were observed when comparing the proteomes of the H^−^A^+^ and H^WT^A^+^ strains, where proteins of most functional categories showed strong differential abundances (Fig. 5b; Fig. S3). These findings strengthen the conclusion that constitutive expression of the WT HtrA in combination with AmyQ production is highly detrimental for the cells, triggering counterselection against the expression of H^WT^. However, it is important to realize that according to our Northern blotting analyses, already at T3, the *htrA* mRNA is not detectable (Fig. 3b). This implies that the rearrangements in the proteome of the H^WT^A^+^ strain compared to the H^−^A^+^ take place already at a very early stage during the culturing of the H^WT^A^+^ strain and/or that the plasmid lacking the promoter region and most of the *htrA* gene is somehow provoking the observed proteomic rearrangements in the cells.

Lastly, at all time points, the H^m^A^+^ strain showed relatively minor rearrangements of the proteome compared to the H^−^A^+^ strain, when compared to the massive proteomic differences observed between the H^−^A^+^ and H^WT^A^+^ strains. In particular, membrane proteins involved in motility were more abundant in the H^m^A^+^ strain than in the H^−^A^+^ strain during all time points of sampling (Fig. 5c; Fig. S3). The strongest effect was seen for proteins involved in arginine biosynthesis and acquisition, where the ArgC, D, F, G, H, and J and CarA and B proteins were more abundant in the H^−^A^+^ strain at T3. However, this situation was reversed by T18 (Fig. S2). This implies that arginine metabolism is influenced by the presence of the proteolytically inactive HtrA. At the later time points during culturing (T6 and T18), proteins related to coping with stress, such as ClpE, CssS, CssR, and LiaH, became more abundant in the H^m^A^+^ strain when compared to the H^−^A^+^ strain. Another important difference between H^−^A^+^ and H^m^A^+^ strains concerned the extracellular proteases AprE, Bpr, and Vpr, which were more abundant in the H^−^A^+^ strain with fold changes of 4.2, 3.5, and 6.7, respectively. Conceivably, this could be one of the reasons why the H^m^A^+^ strain displayed a much higher level of amylase activity than the H^−^A^+^ strain (Fig. 2b; Fig. S2). However, the abundance of these three proteases in the H^WT^A^+^ strain was even lower than observed for the H^−^A^+^ strain (Fig. S4a and c), whereas this had no positive influence on the AmyQ activity (Fig. 2). Therefore, the lower abundance of AprE, Bpr, and Vpr is most likely not a critical factor for achieving the superior yields of active AmyQ with the H^m^A^+^ strain.

### Amylase yield, metabolic activity, and secretion stress during fermentation

**Fig 6:**
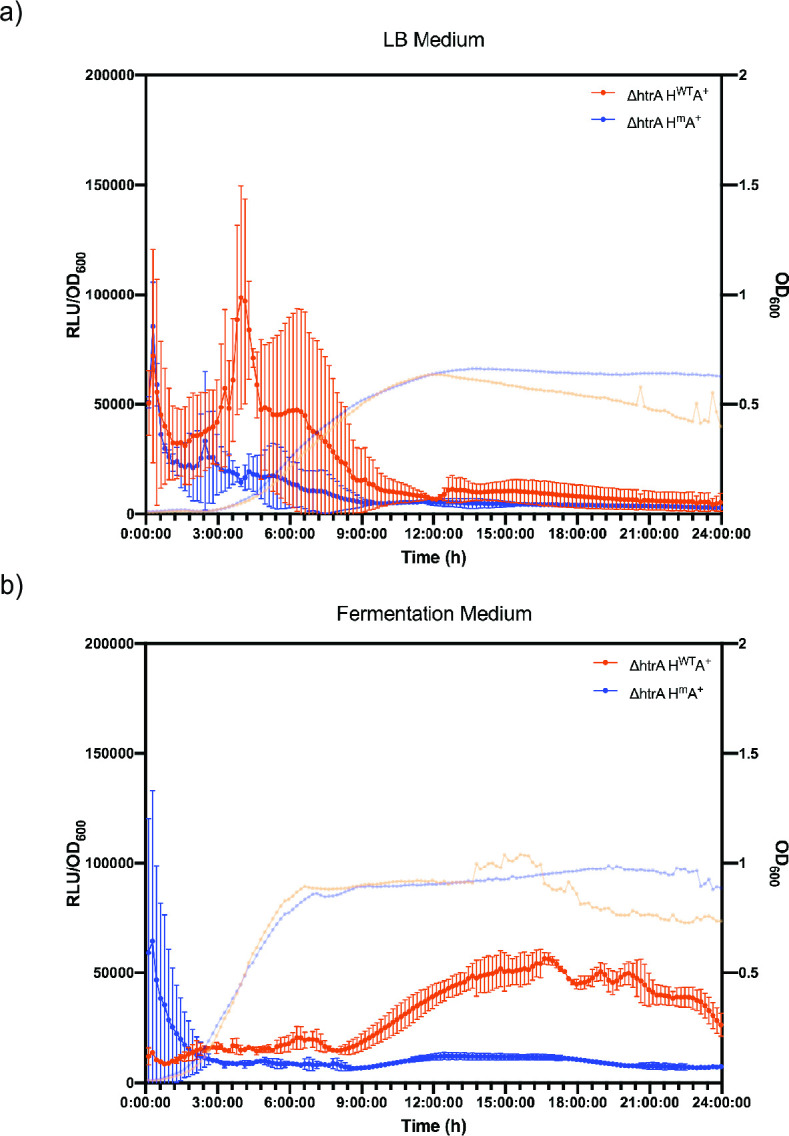
*HtrA* promoter activities of *B. subtilis* strains in lysogeny broth (LB) and fermentation medium (FM). *B. subtilis* strains H^WT^ and H^m^ with (A^+^) or without (A^−^) the plasmid pKTH10 were grown overnight in (a) LB or (b) FM supplemented with antibiotics. Pre-cultures were started from the overnight cultures by diluting 10-fold with (a) LB or (b) FM supplemented with antibiotics. When the pre-cultures reached the exponential growth phase, cultures were diluted to an OD_600_ of 0.05 in (a) LB or (b) FM, and 150-µL aliquots were transferred to a black 96-well plate with clear bottom. The bioluminescence and OD_600_ were measured every 10 min for 24 h in a BioTek Synergy 2 plate reader (37°C, continuous shaking). Real-time measurements of relative light intensity units (RLU)/OD_600_ are indicated on the left *y*-axis and of OD_600_ on the right *y*-axis. Data points and error bars indicating mean values and SEM of three independent experiments are presented.

In a final series of experiments, we aimed at investigating whether co-expression of the mutant HtrA would be beneficial for AmyQ production under industrial fermentation-mimicking conditions. To this end, we employed an optimized fermenter medium (FM). For comparability of the data, we first took a step back and analyzed secretion stress induction with the *lux* reporter system upon microtiter plate cultivation in FM. Surprisingly, the secretion stress profiles recorded during growth in FM were completely different from the secretion stress profiles observed when the bacteria were grown in LB medium (Fig. 6a and b; Fig. S5). In LB, secretion stress was observed starting from the exponential growth phase until the early stationary growth phase (Fig. 1c and d and 6a; Fig. S5b, blue lines), whereas in FM we observed secretion stress induction during the late stationary phase (Fig. 6b; Fig. S5b, red lines). Notably, the strain producing proteolytically inactive HtrA displayed only a marginal secretion stress response in FM. Together, these data show that the growth medium has a profound role in the induction of secretion stress, which has not been reported before.

**Fig 7:**
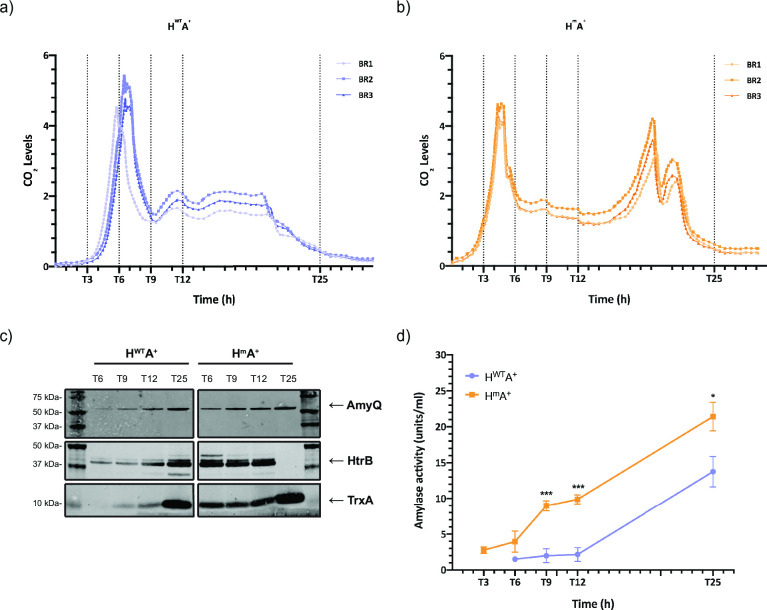
Metabolic activity and AmyQ production by the *B. subtilis* strains during small-scale batch fermentation. *B. subtilis* strains H^WT^A^+^ and H^m^A^+^ were grown overnight in FM supplemented with antibiotics. Subsequently, new overnight cultures were started in a dilution series from 10^−1^ to 10^−10^ in FM. Five hundred-milliliter fermenters were inoculated with exponentially growing cultures from the dilution series with a starting OD_600_ of 0.05. Samples were withdrawn after 3, 6, 9, 12, and 25 h from the start of the main culture. (a and b) CO_2_ levels were measured at the exhaust outlet of the fermenters. Sampling times are indicated with dotted vertical lines. Data from three biological replicates are presented. (c) Samples were standardized to OD_600_ of 2, and the growth medium fraction was obtained by centrifugation. The growth medium fraction was used for trichloroacetic acid precipitation. For visualization of AmyQ levels, the samples were diluted 10-fold. Equal volumes of protein samples were loaded on the gel, and the proteins were separated by LDS-PAGE. AmyQ, HtrB, and TrxA levels were assessed by Western blotting with specific polyclonal antibodies, respectively. The cytoplasmic TrxA protein was used as a marker for cell lysis. (d) Amylase activity in the growth medium fractions of standardized samples was measured by following the amylase activity assay kit instructions (α-Amylase Assay Kit, Megazyme Cat. No. K-CERA). Student’s *t*-test was used to determine significant changes (*0.01 < *P* < 0.05; ***P < 0.001).

To test the performance of the H^m^A^+^ and H^WT^A^+^ strains during fermentation, 0.5-L fermenters were inoculated with bacteria growing exponentially in FM. The batch fermentation process was then carried out for 25 h in the absence of antibiotics, and the metabolic activity of the bacteria was monitored by quantifying the CO_2_ release. Both strains showed an initial peak of metabolic activity after 4–6 h. However, the H^m^A^+^ strain showed two additional peaks of metabolic activity around 18 h after the start of the fermentation (Fig. 7a and b). This was indicative of major differences in cellular metabolism, especially during the later stages of fermentation. During the fermentation, samples were collected for detailed analyses at 3, 6, 9, 12, and 25 h for H^m^A^+^ strain. For the H^WT^A^+^ strain, samples were collected at 6, 9, 12, and 25 h, because this strain displayed a longer lag phase than the H^m^A^+^ strain (Fig. S6).

As a first approach to analyze the fermentation samples, we performed a Northern blotting analysis, in particular, to verify whether the H^WT^A^+^ strain expressed the *htrA* gene. Similar to the shake flask experiments, in the fermentation setup, no *htrA* transcripts were detectable in the H^WT^A^+^ strain at any of the time points of sampling. In contrast, we detected *htrA* transcripts in the H^m^A^+^ strain at all time points (Fig. S7, right panel). This finding is consistent with the notion that there is a strong counterselection against cells that produce both the WT HtrA and AmyQ. On the other hand, in both strains, we detected the *amyQ* transcript at all time points during fermentation, where the H^m^A^+^ strain showed higher *amyQ* transcript levels at T6, T9, and T12 (Fig. S7, left panel).

Importantly, the AmyQ levels in the growth medium samples increased over time as judged by Western blotting (Fig. 7c). Although comparable amounts of the AmyQ protein were secreted by the H^m^A^+^ and H^WT^A^+^ strains at T25, the H^m^A^+^ strain displayed significantly higher extracellular amylase activity levels (Fig. 7d). This implied that the H^m^A^+^ strain produced AmyQ with a higher specific activity than the H^WT^A^+^ strain, which is consistent with the idea that the proteolytically inactive HtrA retains the chaperone activity.

**Fig 8:**
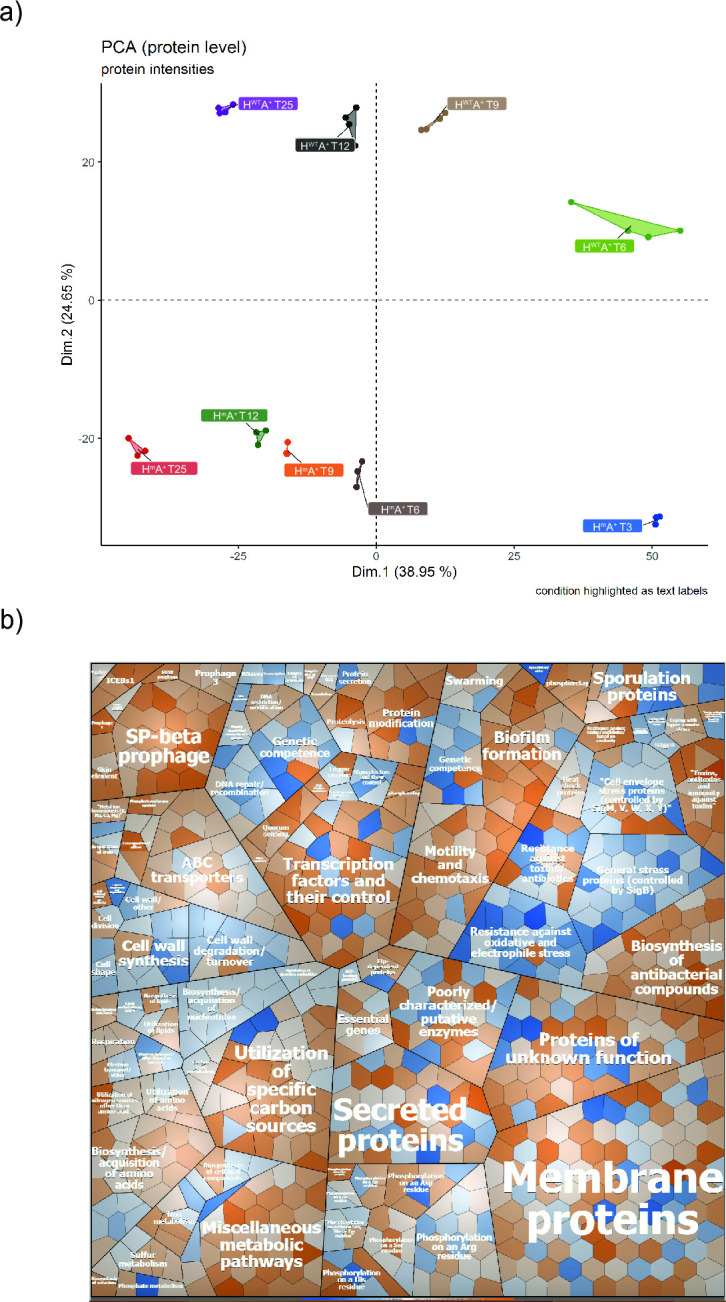
Proteome analysis of *B. subtilis* strains cultured in fermenters. (a) Principal component analysis (PCA) of the LFQ protein intensities. Three biological replicates of each condition are marked with the same color. (b) Voronoi treemap of the quantified proteins clustered per third-level functional category according to the SubtiWiki database. Proteins marked in orange color were more abundant in the H^m^A^+^ strain, and proteins marked in blue color were more abundant in H^WT^A^+^ strain. Increased color intensity correlates with greater differences in protein abundance.

To assess the impact of the proteolytically inactive HtrA on the cellular physiology, we analyzed the proteomes of the H^m^A^+^ and H^WT^A^+^ bacteria collected during the fermentation. PCA revealed that the biological replicates of each strain grouped together in different clusters and that the respective clusters were separated per strain and over time (Fig. 8a). Inspection of the individual protein levels revealed many differences between the H^m^A^+^ and H^WT^A^+^ strains. In particular, this concerned proteins involved in cellular stress responses, as exemplified by proteins controlled by the general stress regulator SigB and proteins involved in the resistance against oxidative and electrophile stress, which were more abundant in the H^WT^A^+^ strain than H^m^A^+^ strain (Fig. 8b; Fig. S8). Conversely, the levels of proteins involved in the utilization of specific carbon sources, like inositol, glucomannan, mannose, and rhamnose, were significantly higher in the H^m^A^+^ strain when compared to H^WT^A^+^ strain. It is also noteworthy that at time points before and after the second peak of metabolic activity displayed by the H^m^A^+^ strain, we observed differences in the abundance of proteins involved in the biosynthesis and acquisition of amino acids, as well as “protein utilization.” This included the extracellular proteases AprE, Bpr, and Vpr, which were more abundant in the H^m^A^+^ than in the H^WT^A^+^ strain at T25 (Fig. S4d to f). Altogether, it seems likely that the observed changes at the proteome level account for the second and third peaks of metabolic activity observed during fermentation of H^m^A^+^ strain. The bigger picture shows that the H^m^A^+^ strain activates more metabolic pathways to sustain growth at the late stages of fermentation, while keeping a lower level of stress-related proteins. This could explain the superior performance of this strain during fermentation in terms of high-quality AmyQ production. This was also the case at T25, despite elevated levels of AprE, Bpr, and Vpr, which implies that a chaperone-like activity of the proteolytically inactive mutant HtrA could help to prevent AmyQ degradation by promoting the folding of the translocated AmyQ into its protease-resistant fully folded state.

### Conclusion

In conclusion, this study highlights the beneficial contribution of proteolytically inactive HtrA to the secretion of the heterologous protein AmyQ by *B. subtilis* even upon upscaling. Under all tested conditions, the proteolytically inactive HtrA promoted AmyQ secretion, while similar constitutive expression of the proteolytically active WT HtrA triggered strong counterselection. In addition, we show that this mutant HtrA can mitigate potentially detrimental secretion stress responses. Our present observations also explain why previous attempts to harness the WT HtrA for improved protein production in *B. subtilis* have failed, as its constitutive co-expression with AmyQ turned out severely detrimental for the bacteria. Lastly, we conclude that the H^m^A^+^ strain presented in this study can be readily cultured under industrial fermentation-mimicking conditions and that it produces high levels of enzymatically active amylase. Altogether, these findings imply that the expression of proteolytically inactive HtrA leads to a remodeling of cellular stress responses, which is beneficial for obtaining high yields of amylase with optimal specific activity. It will be an important objective for future studies to investigate whether the secretion of other proteins will also benefit from co-expression of proteolytically inactive HtrA. This may be aided by introducing the HtrA-inactivating Ser260Ala mutation into a chromosomal copy of the *htrA* gene.

## MATERIALS AND METHODS

### Bacterial strains

All the bacterial strains used in this study are listed in Table 1.

**TABLE 1:** Strains used in this study

Strain	Characteristics	Reference
***Bacillus subtilis***		
168	*trpC2*	Kunst et al. (28)
168 *htrA*::*spec*	*trpC2*, HtrA-deficient background, Spec^R*a*^	Zweers et al. (29)
168 *sacA*::*plux_phtrA*	*trpC2*, P_htrA_ *lux* reporter strain, Cm^R*a*^	This study
168 Δ*yqfD*	*trpC2*, *yqfD* marker-less deletion	This study

^*a*^ Spec^R^, spectinomycin resistance; Cm^R^, chloramphenicol resistance.

### Growth conditions

*E. coli* strains were grown in LB medium at 37°C with shaking at 250 rpm. *B. subtilis* strains were grown in LB medium or fermentation medium (FM) at 37°C with shaking at 250 rpm for unbaffled flasks or 180 rpm for baffled shake flasks. FM consisted of 2% yeast extract, 2.5% peptone, 1% saccharose, 1% NaH_2_PO_4_.H_2_O, 1% Na_2_HPO_4_.H_2_O, and 0.5% potato dextrose. For selection of transformed *E. coli* strains, media were supplemented with ampicillin (100 µg/mL), kanamycin (50 µg/mL), erythromycin (100 µg/mL), or chloramphenicol (10 µg/mL) when necessary. For selection of transformed *B. subtilis* strains, media were supplemented with spectinomycin (100 µg/mL), kanamycin (20 µg/mL), erythromycin (2 µg/mL), or chloramphenicol (5 µg/mL) when necessary.

### Batch fermentation

*B. subtilis* cultures were inoculated in baffled shake flasks containing FM and antibiotics overnight at 37°C with shaking at 180 rpm. The overnight cultures were serially diluted in FM and incubated overnight at 37°C with shaking at 180 rpm. Exponentially growing cultures were used to inoculate 0.5-L fermenters containing FM at a final OD_600_ of 0.05. Temperature, agitation, and aeration rate were maintained at 37°C, 1,200 rpm, and 200 ccm, respectively. The pH was initially set at 6.9 and adjusted to 7.1 ± 0.2 with 12.5% NH_3_ or 12.5% H_2_SO_4_. CO_2_, and pH levels were measured throughout the fermentation process.

### Construction of plasmids and strains

All plasmids used in this study are listed in Table 2.

**TABLE 2:** Plasmid used in this study

Plasmid	Description	Reference
pUB110	Km^R*a*^	Gryczan et al. (30)
pKTH10	pUB110 derivative encodes the α-amylase AmyQ from *B. amyloliquefaciens*, Km^R^	Palva (31)
pHB201_WT HtrA	Constitutive expression of WT HtrA of *B. subtilis*, Em^R*a*^, Cm^R*a*^	This study
pHB201_mut HtrA	Constitutive expression of proteolytically inactive HtrA of *B. subtilis*, Em^R^, Cm^R^	This study
pBS3Clux_phtrA	Integrative plasmid with P*htrA*-directed expression of the *luxABCDE*-operon of *P. luminescens*, Cm^R^, Amp^R*a*^	This study

^*a*^ Amp^R^, ampicillin resistance; Cm^R^, chloramphenicol resistance;Em^R^, erythromycin resistance; Km^R^, kanamycin resistance.

Sequences of all primers used for cloning are listed in Table S2. pBS3Clux_phtrA was constructed by amplifying the *htrA* promoter sequence from the genome of *B. subtilis* IIG-Bs1 and by introducing EcoRI and PstI restriction sites with PCR using primers P1 and P2. The PCR fragment and pBS3Clux vector were cleaved with EcoRI and PstI, and, subsequently, the resulting DNA fragments were ligated using T4 DNA ligase, which led to pBS3Clux_phtrA. For genomic integration of this secretion stress sensor, pBS3Clux_phtrA was cleaved with ScaI and the linearized vector was used to transform the *B. subtilis* 168 and *B. subtilis* 168 *htrA*::spec strains.

To produce plasmid systems expressing the WT or the proteolytically inactive version of HtrA, the WT *htrA* gene was amplified via PCR using P2 and P3 from the genomic DNA of the *B. subtilis* IIG-Bs1 strain, and restriction sites for EcoRI and KpnI were introduced at the ends. A mutated *htrA* gene fragment was constructed using overlap extension PCR, and restriction sites for EcoRI and KpnI were introduced at the ends using the primers P3, P4, P5, and P6. The WT *htrA* gene and mutated *htrA* gene fragments, as well as the pHB201 vector, were cleaved with EcoRI and KpnI. The resulting DNA fragments were ligated to the vector using T4 DNA ligase. After transformation, *E. coli* cells were grown overnight on LB agar plates containing 1-mM IPTG, 0.2-mg/mL X-gal, 100-µg/mL erythromycin, and 10-µg/mL chloramphenicol. White colonies were selected for colony PCR, and positive colonies were sent for sequencing. After sequence confirmation, pHB201_WT HtrA and pHB201_mut HtrA were used to transform *B. subtilis* 168 *sacA*::p*lux*_p*htrA htrA*::spec.

### Bioluminescence assay

*B. subtilis* strains were grown in LB medium supplemented with antibiotics at 37°C with shaking at 250 rpm for 16 h. Overnight cultures were diluted 10-fold in fresh LB without addition of any antibiotics and incubated at 37°C with shaking at 250 rpm for 3 h. After the incubation, the optical density at 600 nm (OD_600_) was measured, and each strain was standardized to 0.05 OD_600_ units. One hundred fifty microliters of each culture was transferred to a 96-well plate. Three technical replicates were made for each sample. A BioTek Synergy 2 plate reader was used to measure OD_600_, and luminescence values every 10 min for 24 h. During the experiment, the plate was kept at 37°C and shaken in between measurements.

### LDS-PAGE and Western blotting

Cultures were centrifuged to separate cells and growth medium fractions. Ten percent (wt/vol) trichloroacetic acid was used to precipitate the proteins in the growth medium. Precipitated proteins were resuspended in lithium dodecyl sulfate (LDS) gel loading buffer (Life Technologies). For the cell pellet, LDS gel loading buffer and glass beads were added, and bead beating was applied for cell disruption using a Precellys 24 tissue homogenizer. All samples were incubated at 95°C for 10 min. Proteins in the samples were subsequently separated on 10% pre-cast Bis-Tris NuPage gels (Invitrogen). For visualization of the separated proteins, gels were stained with SimplyBlue SafeStain (Life Technologies).

For Western blotting, proteins separated by LDS-PAGE were transferred onto a nitrocellulose membrane by semi-dry blotting (Amersham Protran 0.45 µm, GE Health Care Sciences). Membranes were blocked with 5% (wt/vol) skim milk and washed with phosphate-buffered saline plus Tween20 (PBS-T). The membranes were then incubated with specific polyclonal rabbit antibodies against AmyQ, HtrB, or TrxA (1:5,000) and incubated for 1 h at room temperature. After the incubation, the membranes were washed with PBS-T and incubated with a secondary goat-anti rabbit IgG IRDye800 CW (1:5,000; LI-COR). Finally, the membranes were washed three times with PBS-T and twice with PBS. Visualization was performed using an Amersham Typhoon Biomolecular imager.

### Amylase activity assay

The α-Amylase Assay Kit (Ceralpha Method) from Megazyme was used to determine α-amylase enzymatic activity in growth medium fractions of different strains. Briefly, cultures were standardized to the same OD_600_ units in 2 mL and centrifuged at maximum speed for 2 min. Ten microliters of the supernatant was transferred to 490 µL of Buffer B containing 0.1-M maleic acid, 0.1-M NaCl, 2-mM CaCl_2_ dihydrate, and 0.01% NaN_3_, to obtain the “original extract.” This original extract was used to obtain 1:50 and 1:500 dilutions using Buffer B. In separate tubes, 100 µL of substrate was prepared. Both the substrate and diluted samples were incubated at 40°C for 5 min. Then, 100 µL of each dilution was added on the substrate, and the mixture was incubated for 10 min at 40°C. The reaction was stopped using stopping reagent from the kit supplier, and 1 mL of the mixture was transferred into cuvettes. OD_400_ was measured using a Cary 60 UV-Vis spectrophotometer. Activity was calculated according to the kit manufacturer’s directions.

### RNA extraction and Northern blotting

Total RNA was prepared by acid-phenol extraction after mechanical cell disruption as described previously (32). For Northern blot analysis, 4 µg of total RNA per sample was separated under denaturing conditions in 1.2% agarose gels prepared by mixing 1.2-g agarose, 74-mL water, 10-mL 10× MOPS, 10-mL formaldehyde (37%), and 5-µL ethidium bromide. After electrophoretic separation with 1× MOPS as running buffer, RNA quality and equal loading were controlled by visualization of the 16S and 23S rRNA under UV light. Subsequently, the RNA was transferred by vacuum blotting onto a positively charged nylon membrane (Roche Diagnostic GmbH, Mannheim, Germany) and cross-linked (120 mJ/cm^2^; Stratalinker UV Crosslinker 1800; Agilent Technologies, Santa Clara, CA, USA). The membranes were prehybridized for 1 h at 68°C and hybridized overnight at 68°C with continuous rotation in 15 µL of hybridization solution containing 50% formamide, 5× SSC, 0.02% SDS, 0.1% N-lauroyl sarcosinate, 2% blocking reagent, and 1 µg of digoxygenin (DIG)-labeled RNA probe. DIG-labeled RNA probes were synthesized by *in vitro* transcription with T7 RNA polymerase and gene-specific PCR products as template. The respective primer sequences are listed in Table S3. After washing, the membranes were incubated for 30 min at room temperature with 1:10,000 diluted anti-DIG-AP Fab antibody and washed again. Then, the pH was adjusted to 9.5, and the chemiluminescent substrate Tropix CDP-Star was added. Chemiluminescence signals were detected using a ChemoCam Imager (Intas Science Image Instruments, Göttingen, Germany).

### Sample preparation for MS

Sixteen OD_600_ units of bacterial culture were harvested. Tubes were briefly immersed in liquid nitrogen to stop the metabolism of the cells. Cells were pelleted by centrifugation at 4,000 rpm for 2 min at 4°C. Cells were resuspended in 1 mL of 20-mM HEPES and subsequently centrifuged at 40,00 rpm for 2 min at 4°C. The supernatant was discarded, and cell pellets were frozen in liquid nitrogen. All samples were stored at −80°C until sample preparation.

Cell pellets were resuspended in 100 µL of 20-mM HEPES with 5% SDS. Cells were disrupted in a Sartorius Micro Dismembrator for 3 min at 2,600 rpm. Samples were resuspended in 400 µL of 20-mM HEPES, which was preheated to 95°C and transferred into low-binding pre-lubricated 1.5-mL tubes. Tubes were shaken for 1 min at 95°C; 4-mM MgCl_2_ and 0.005 U/µL of benzonase (final concentration) were added to each sample. Tubes were vortexed briefly and kept in an ultrasonic bath for 5 min. After centrifugation at maximum speed for 30 min, the supernatants were transferred to a new tube to be used for Pierce BCA Protein Assay Kit (Thermo Fisher Scientific, MA, USA) and digestion.

Protein preparation was carried out using a paramagnetic bead technology, termed single-pot solid-phase-enhanced sample preparation (SP3) (33, 34). Each sample was standardized to 4 µg in 20 µL by dilution with 20-mM HEPES. A final concentration of 3.3 µg/µL bead stock μL of a (1:1) bead combination mix of hydrophilic and hydrophobic Cytiva Sera-Mag SpeedBeads Carboxyl-Magnet-Beads (Thermo Fisher Scientific, MA, USA) was added to each sample as described by (33, 33). Next, 100% acetonitrile was added to each tube to a final concentration of 70%. Tubes were shaken at room temperature for 18 min at 1,400 rpm. A magnetic rack was used to attach the beads to the side of the tube. Subsequently, after bead sedimentation of 2 min, the supernatant was removed, and the beads were washed with 70% ethanol twice with bead sedimentation of 2 min and removal of supernatants. Lastly, the beads were washed with 100% acetonitrile and left to air-dry.

For on-bead trypsin digestion, 8 µL of 20-ng/µL trypsin (Promega, WI, USA) and 2 µL of ammonium bicorbante buffer were added to each tube, and the tubes were incubated at 37°C for 16 h. The digestion was stopped by addition of acetonitrile to a final concentration of 95% (vol/vol). Tubes were shaken at room temperature for 18 min, and by using a magnetic rack, the supernatant was discarded. One hundred eighty microliters of 100% acetonitrile was added, and the tubes were vortexed. After removal of the supernatant, the beads were air-dried. To elute the proteins, 10 µL of 2% DMSO was added to each tube, and the beads were resuspended by scraping with a pipet tip. The tubes were kept for 5 min in an ultrasonic bath, and then 10-µL 2× Buffer A (4% acetonitrile and 0.2% acetic acid) was added to each MS insert. Finally, 10 µL of the supernatant was added to each MS insert.

### Mass spectrometric DIA analyses

For LC-MS/MS analyses, tryptic peptide solutions were separated on an Ultimate 3000 nano-LC system (Thermo Fisher Scientific, MA, USA) and analyzed on a Q Exactive HF mass spectrometer in data-independent acquisition (DIA) mode. For further details on instrument setup and methods for DIA mode, see Tables S4 and S5.

The analysis of DIA data was performed using Spectronaut version 16 in a direct DIA approach (BiognoSYS, Schlieren, Switzerland), R (R version 4.2.0, 2022–04-22 ucrt), and tidyverse package (version:1.3.1). Settings for spectronaut analysis are specified in Table S6. In brief, a direct DIA approach was used based on a database search against a UniProt/SwissProt database restricted to entries from *B. subtilis* (version 20210705) with the addition of AmyQ (P00692), chloramphenicol acetyltransferase (P00485), kanamycin nucleotidyltransferase (P05057), and rRNA adenine N-6-methyltransferase (P13956). The trypsin/P digest rule was used with a maximum number of two missed cleavages, and methionine oxidation was selected as variable modification. Normalization of data was carried out in Spectronaut based on a local regression model (35).

Statistic DIA-MS data analysis was performed using R according to the following steps: (i) replacement of 0 intensity values by half-minimal intensity values from the whole data set; (ii) removal of methionine-oxidized peptides; (iii) calculation of sum over ions per sample and peptide to generate peptide intensity data; and (iv) generation of maxLFQ protein intensity data by using the maxLFQ algorithm implemented in the iq R package (version 1.9.6) followed by a global median normalization. The PCA for peptide and protein level was performed using the factomine R package (version 2.4). Only proteins identified with at least two peptides were considered for quantitative analyses. A ROPECA approach reproducibility-optimized peptide change averaging test (ROTS) statistics was used for statistical analyses (36).

Voronoi treemaps were made using the Paver software (Decodon GmbH) by using the SubtiWiki database for functional categories and regulons.

## Data Availability

The raw files from the MS analyses have been deposited in MassIVE (https://massive.ucsd.edu) under MSV000091841. Supplementary voronoi treemaps based on regulons and fuctional categories have been deposited on Zenodo with the following https://doi.org/10.5281/zenodo.7868715 and https://doi.org/10.5281/zenodo.7868846, respectively.

## SUPPLEMENTAL MATERIAL

Supplemental file 1 spectrum.01778-23-s0001.pdf Fig. S1 to S8.

Supplemental file 2 spectrum.01778-23-s0002.xlsx Table S1.

Supplemental file 3 spectrum.01778-23-s0003.pdf Tables S2 to S6.

Supplemental file 4 spectrum.01778-23-s0004.xlsx Table S7.

ASM does not own the copyrights to Supplemental Material that may be linked to, or accessed through, an article. The authors have granted ASM a non-exclusive, world-wide license to publish the Supplemental Material files. Please contact the corresponding author directly for reuse.
