# Supplementary material for: Enhancing bacterial fitness and recombinant enzyme yield by engineering the quality control protease HtrA of Bacillus subtilis
Source: Microbiol Spectr. 2023 Oct 11;11(6):e01778-23. doi: 10.1128/spectrum.01778-23 (PMC10715036; doi:10.1128/spectrum.01778-23)
Supplement: Supplemental file 1 — Fig. S1 to S8. [file spectrum.01778-23-s0001.pdf]

Supplementary Figures

Enhancing bacterial fitness and recombinant enzyme yield by engineering the quality control protease HtrA of *Bacillus subtilis*

Ayşegül Öktem[a], David Núñez-Nepomuceno[b], Borja Ferrero-Bordera[c], Jonathan Walgraeve[d], Michael Seefried[d], Manuela Gesell-Salazar[b], Leif Steil[b], Stephan Michalik[b], Sandra Maaß[c], Dörte Becher[c], Ulrike Mäder[b], Uwe Völker[b#], Jan Maarten van Dijl[a#*]

[#]Equal contributions

[a] Department of Medical Microbiology, University of Groningen, University Medical Center Groningen, The Netherlands

[b] Interfaculty Institute for Genetics and Functional Genomics, University Medicine Greifswald, Greifswald, Germany

[c] Department of Microbial Proteomics, Institute of Microbiology, University of Greifswald, Greifswald, Germany

[d] Molecular Biology department, AB Enzymes, Darmstadt, Germany

*Correspondence: Jan Maarten van Dijl, University Medical Center Groningen, Department of Medical Microbiology, Hanzeplein 1, 9700RB Groningen, the Netherlands, tel. +31-50-3615187, e-mail: j.m.van.dijl01@umcg.nl

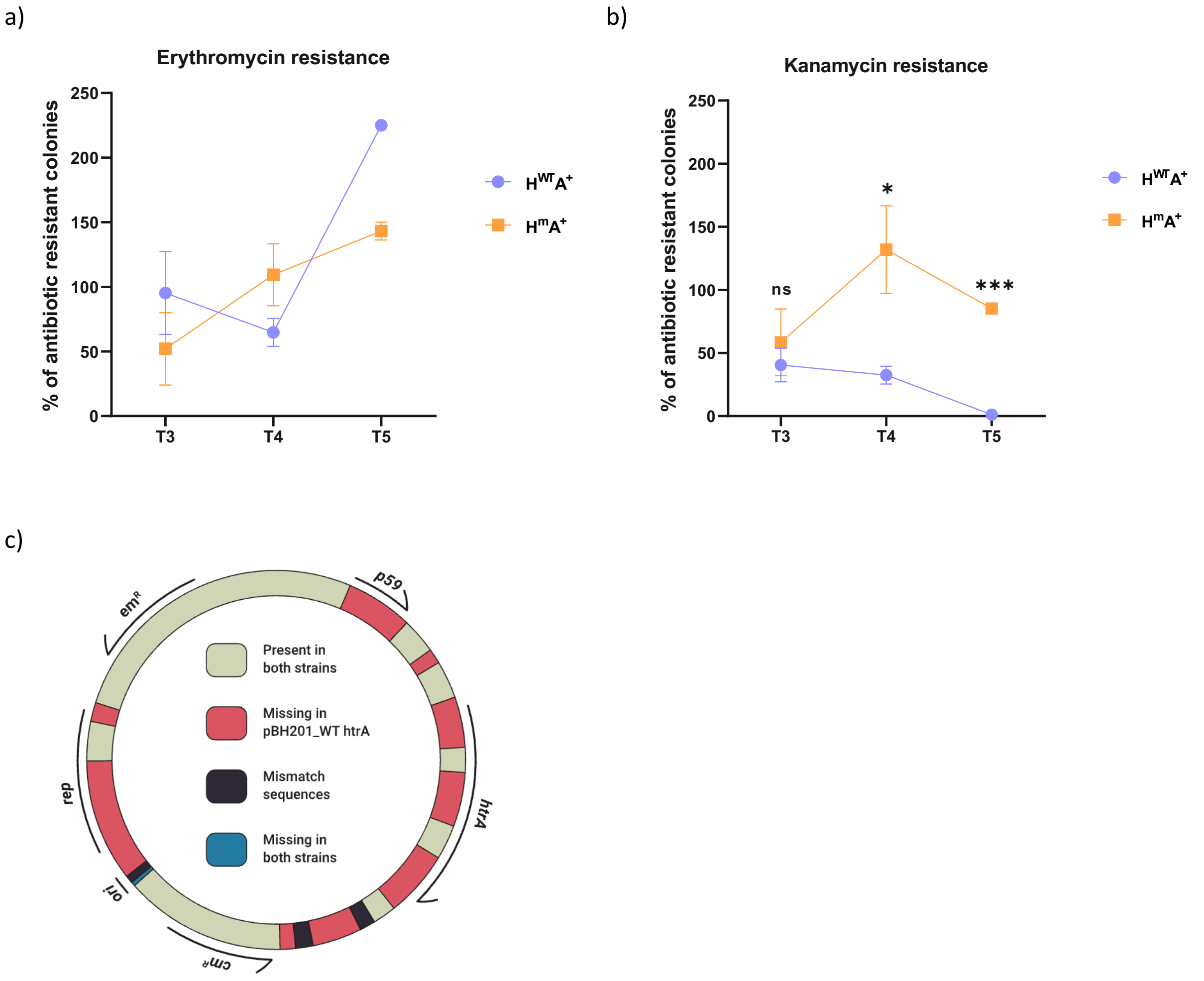

Supplementary Figure S1. Plasmid loss in $H^{WT}A^{+}$ strain. (a-b) $H^{WT}A^{+}$ and $H^{m}A^{+}$ strains were incubated overnight in LB supplemented with antibiotics. A new culture was started from the overnight cultures in LB without antibiotics. Samples from the cultures were plated on LB agar plates without antibiotic, erythromycin (2 µg/mL) or kanamycin (20 µg/mL). Erythromycin was used to select the colonies containing the plasmid pHB201 and kanamycin was used to select the colonies containing the plasmid pKTH10. The total colony count was determined by counting the colonies on the plates without antibiotic. Accordingly, the percentage of antibiotic resistant colonies was calculated by the ratio of colonies on antibiotic containing plate and total colony count multiplied. Student's t-test was used to determine significant changes (ns: not significant, *: $0.01<p<0.05$, ***: $p<0.001$). (c) Cartoon representation of the sequencing results of the plasmids pHB201_WT htrA and pHB201_mut htrA. $H^{WT}A^{+}$ and $H^{m}A^{+}$ strains were streaked on LB agar supplemented with antibiotics. A single colony was selected per strain for Illumnina sequencing. Sequence alignment was used to determine DNA sequences missing in one or both strains in respect to the reference sequence.

a)

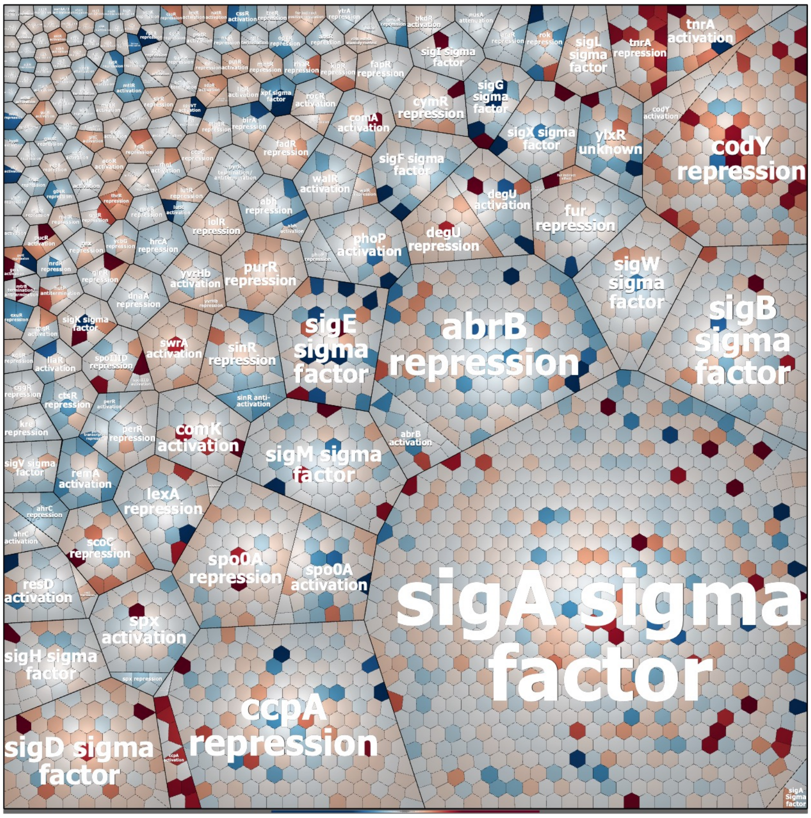
sigA sigma factor
abrB repression
sigB sigma factor
codY repression
ccpA repression
sigE sigma factor
sigM sigma factor
sigD sigma factor
sigW sigma factor
spo0A repression
spo0A activation
comK activation
lexA repression
spx activation
sigH sigma factor
tnrA activation
sigL sigma factor
tnrA repression
sigG sigma factor
sigX sigma factor
ylxR unknown
sigF sigma factor
cymR repression
comA activation
degU activation
degU repression
fur repression
phoP activation
purR repression
sinR repression
swrA activation
resD activation
scoC repression
remA activation
sigI sigma factor
fapR repression
fadR repression
walR activation
yvrHb activation
perR repression
sigV sigma factor
abrB activation
sinR anti-activation
sigK sigma factor

b)

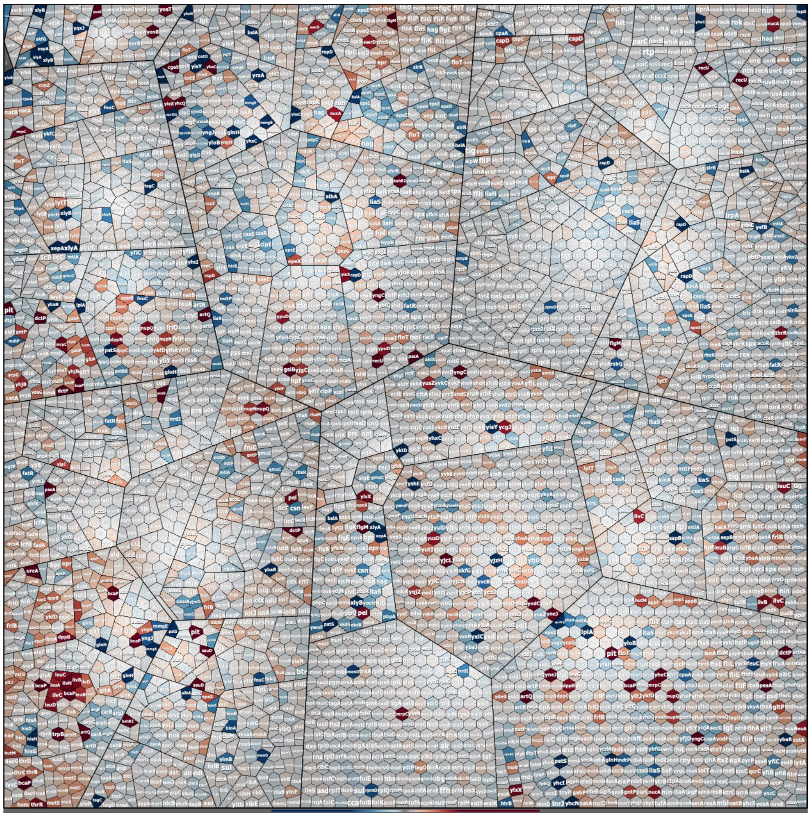

c)

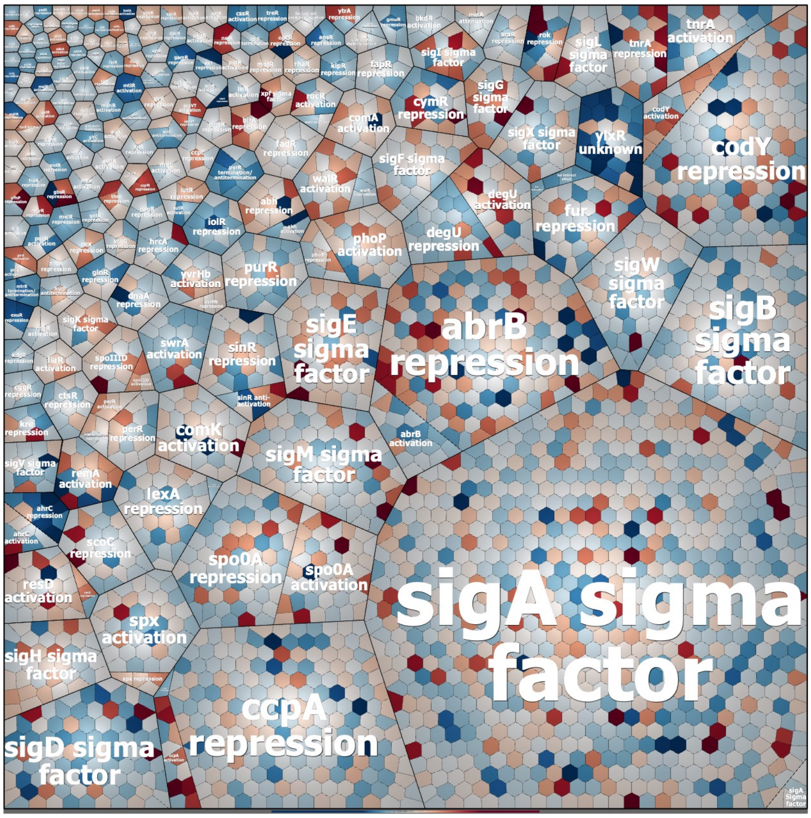

Supplementary Figure S2. Voronoi treemaps of the quantified proteins clustered (a) per regulons for H^-A^- vs H^-A^+ at T3 (b) fifth level (L5) of functional categories based on Subtiwiki database for H^-A^- vs H^-A^+ at T18 and (c) per regulons for H^-A^- vs H^-A^+ at T18. Proteins represented with red color are more abundant in H^-A^- and proteins represented with blue color are more abundant in H^-A^+ strain. The complete set of Voronoi treemaps based on regulons and functional categories based on Subtiwiki database can be accessed at https://doi.org/10.5281/zenodo.7868715 and https://doi.org/10.5281/zenodo.7868846 respectively.

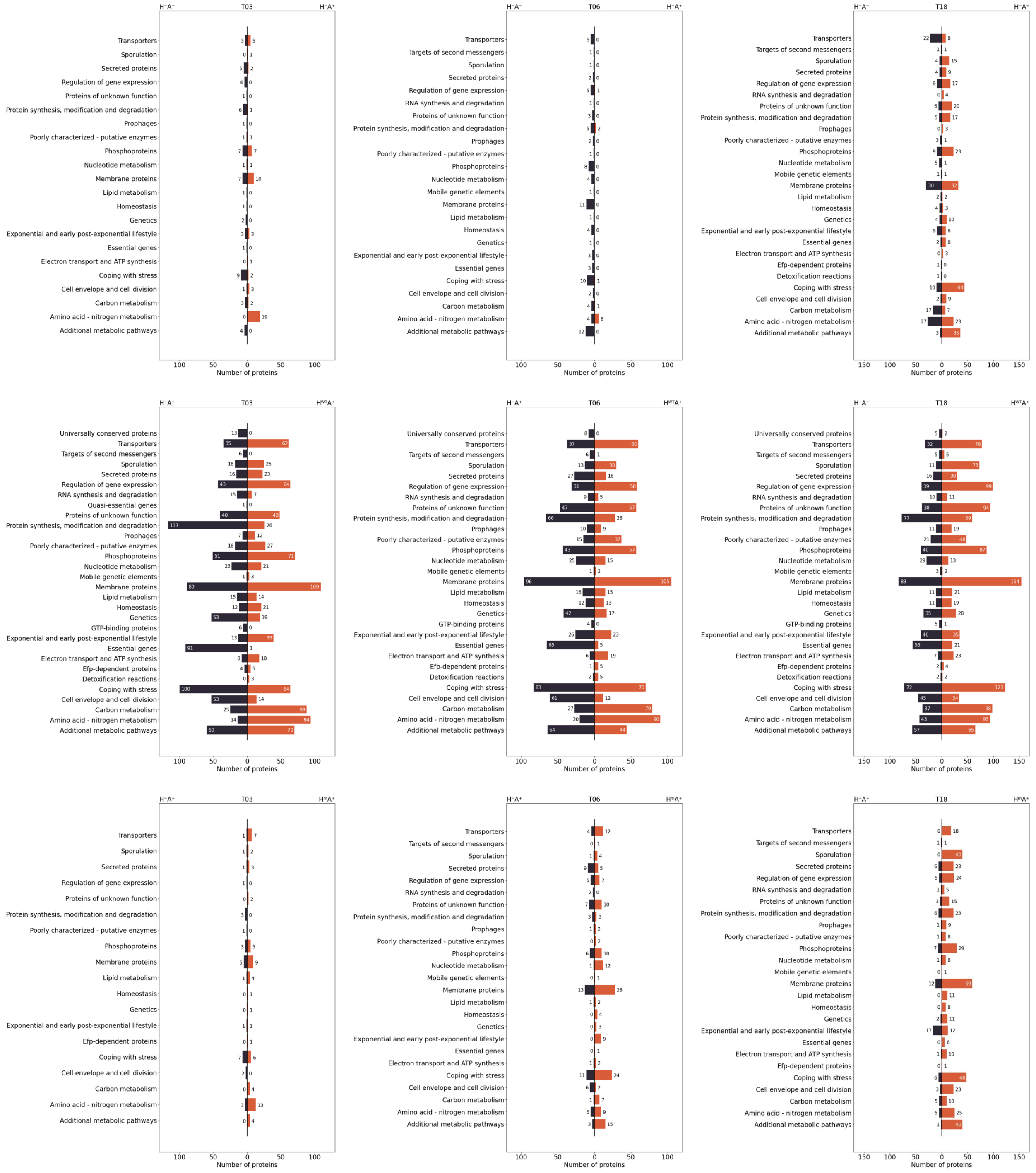

Supplementary Figure S3. Number of significantly changed proteins (p<0.05, fold change>1.5) corresponding to Subtiwiki functional categories in T3, T6 and T18 samples.

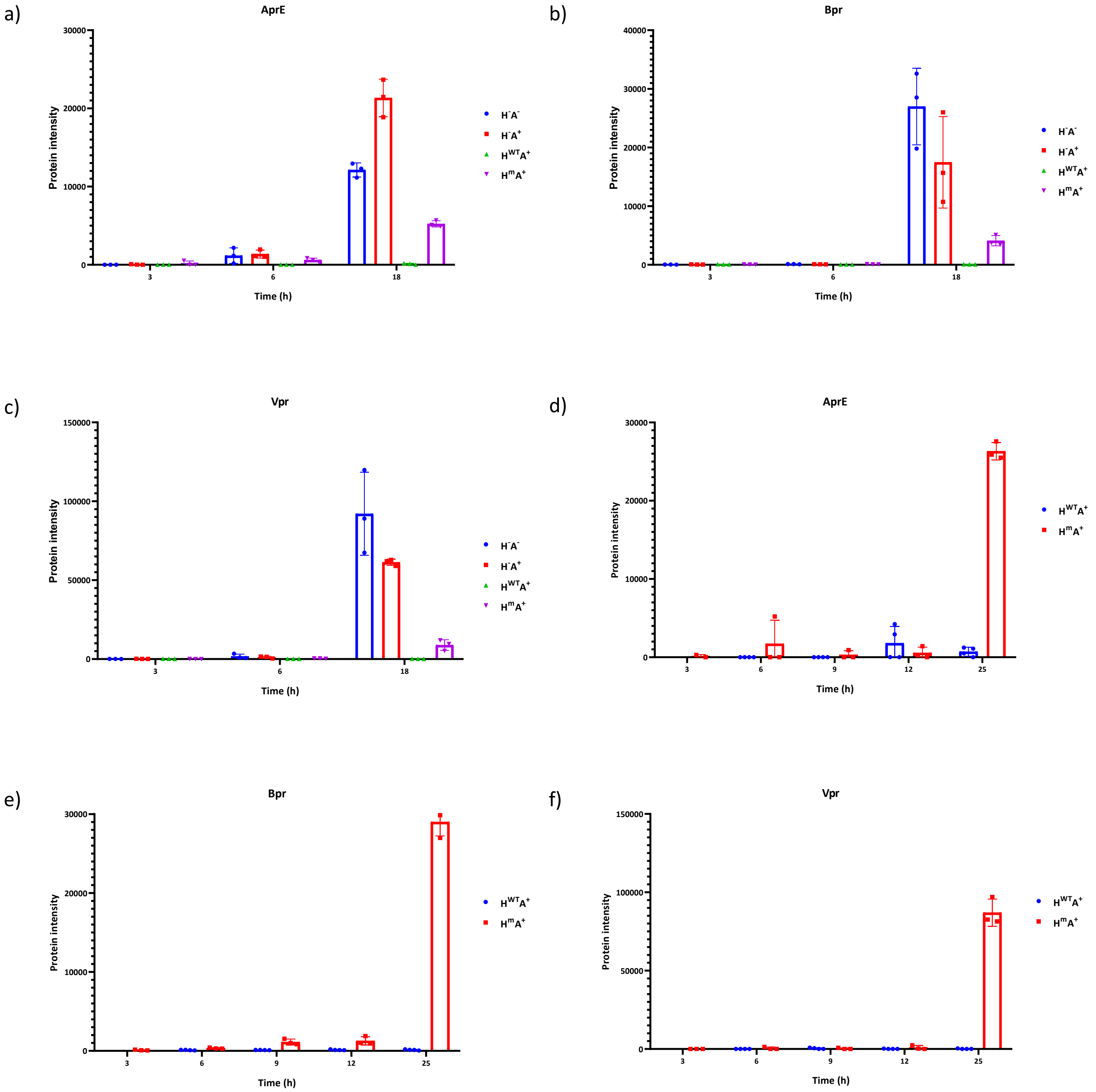

Supplementary Figure S4. Protein intensities of AprE, Bpr and Vpr in (a-c) shake flask samples and (d-f) fermenter samples.

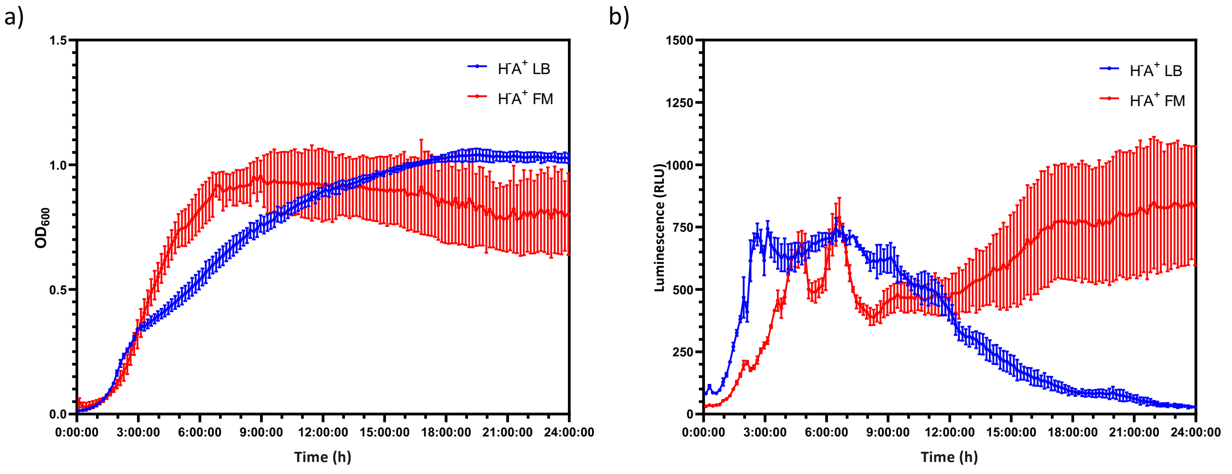

Supplementary Figure S5. *htrA* promoter activity in the H^-A^+ strain. Exponentially growing cells were diluted to OD_{600} of 0.05 in LB or FM and 150 µL aliquots were transferred to a black 96-well plate with clear bottom (Thermo Scientific). The luminescence and OD_{600} were measured every 10 min for 24 h in a Biotek Synergy2 plate reader (37 °C, continuous shaking). Blue lines represent the cells grown in LB and red lines represent cells grown in FM. (a) OD_{600} measurements. (b) Luminescence measurements. Data points and error bars indicating the mean values and SD of 3 independent experiments are presented.

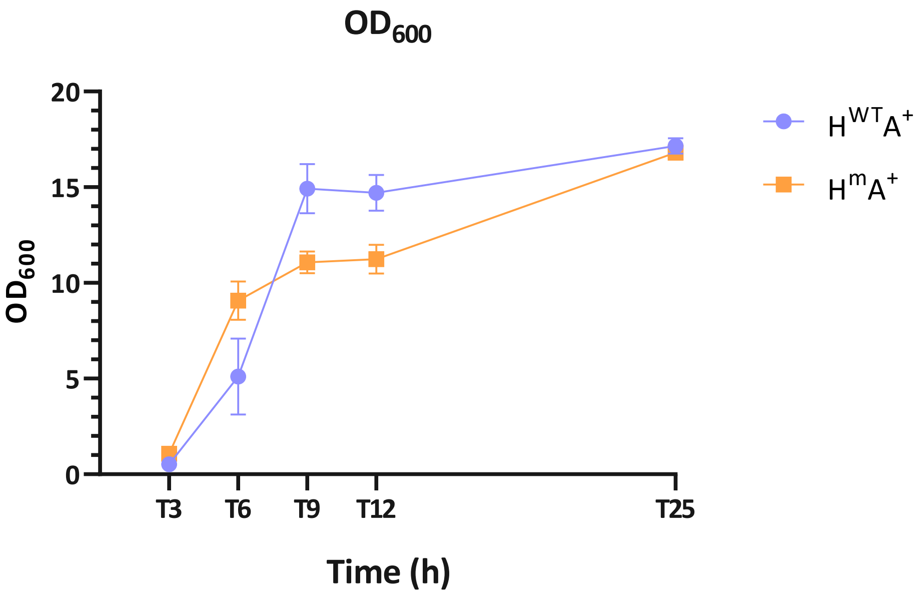

Supplementary Figure S6. OD$_{600}$ values at the time of sample collection from fermenters.

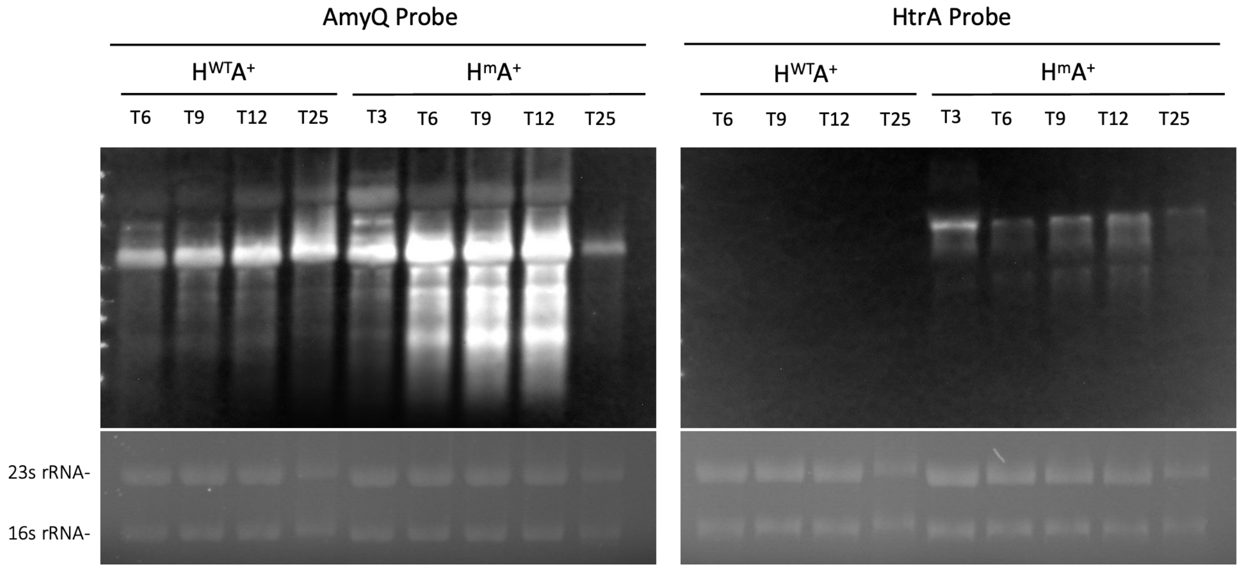

Supplementary Figure S7. Northern blot of fermenter samples. 16 OD units of bacterial culture were harvested, and RNA extraction was carried out. 4 µg of total RNA were loaded in each well. (c) amyQ and (d) htrA transcripts were detected with digoxygenin-labelled antisense RNA probes. On the bottom the rRNA was visualized as loading control.

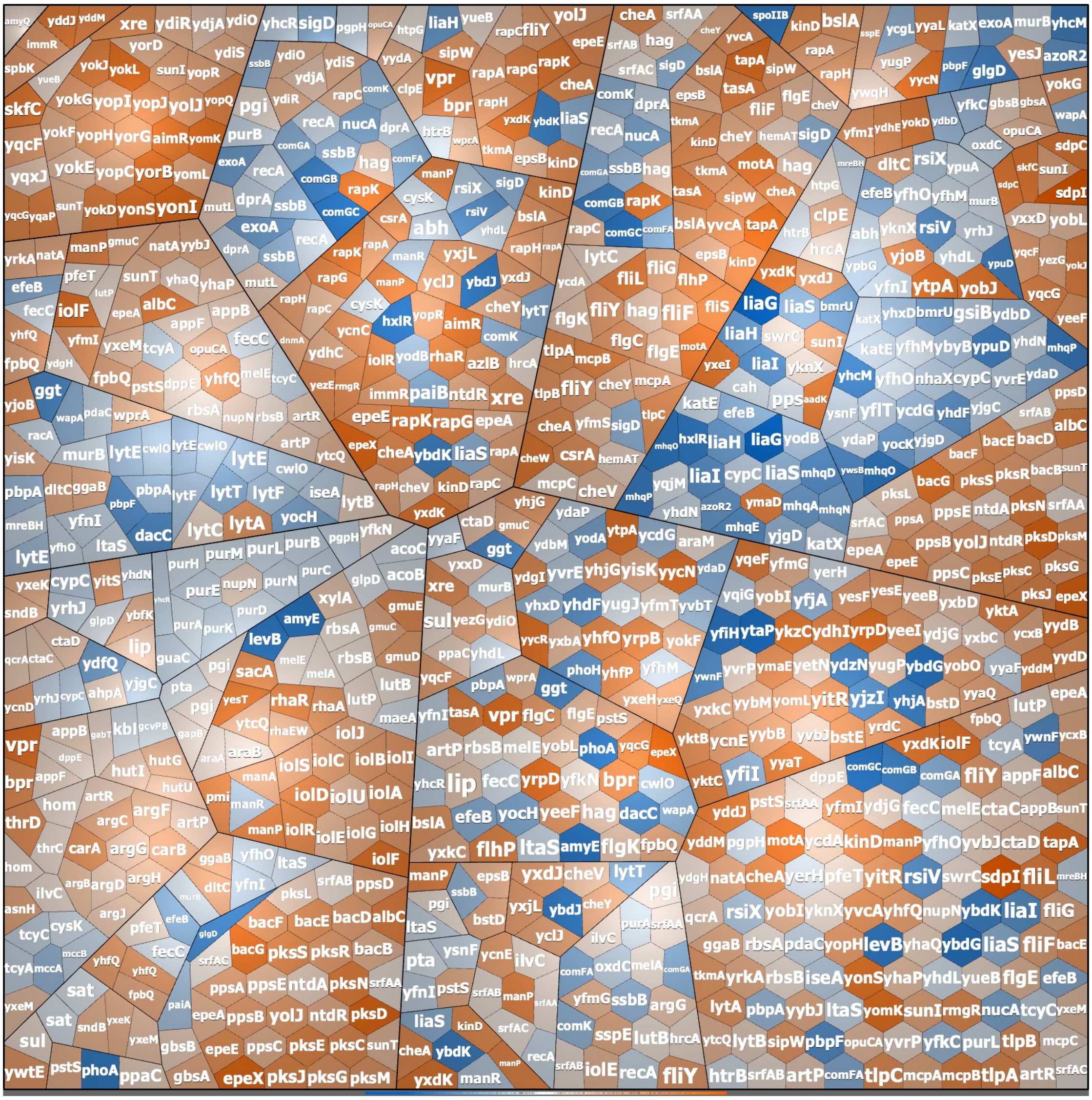

Supplementary Figure S8. Proteome analysis of fermenter samples. Voronoi treemaps of the quantified proteins clustered per fifth level of functional category according to the SubtiWiki database. Proteins with orange color are more abundant in $H^{m}A^{+}$ strain and proteins with blue color are more abundant in $H^{WT}A^{+}$ strain. Increased colour intensity correlates with greater differences in protein abundance.
