# Supplementary material for: Enhancing bacterial fitness and recombinant enzyme yield by engineering the quality control protease HtrA of Bacillus subtilis
Source: Microbiol Spectr. 2023 Oct 11;11(6):e01778-23. doi: 10.1128/spectrum.01778-23 (PMC10715036; doi:10.1128/spectrum.01778-23)
Supplement: Supplemental file 3 — Tables S2 to S6. [file spectrum.01778-23-s0003.pdf]

Supplementary Tables

Enhancing bacterial fitness and recombinant enzyme yield by engineering the quality control protease HtrA of *Bacillus subtilis*

Ayşegül Öktem[a], David Núñez-Nepomuceno[b], Borja Ferrero-Bordera[c], Jonathan Walgraeve[d], Michael Seefried[d], Manuela Gesell-Salazar[b], Leif Steil[b], Stephan Michalik[b], Sandra Maaß[c], Dörte Becher[c], Ulrike Mäder[b], Uwe Völker[b#], Jan Maarten van Dijl[a#*]

[#]Equal contributions

[a] Department of Medical Microbiology, University of Groningen, University Medical Center Groningen, The Netherlands

[b] Interfaculty Institute for Genetics and Functional Genomics, University Medicine Greifswald, Greifswald, Germany

[c] Department of Microbial Proteomics, Institute of Microbiology, University of Greifswald, Greifswald, Germany

[d] Molecular Biology department, AB Enzymes, Darmstadt, Germany

*Correspondence: Jan Maarten van Dijl, University Medical Center Groningen, Department of Medical Microbiology, Hanzeplein 1, 9700RB Groningen, the Netherlands, tel. +31-50-3615187, e-mail: j.m.van.dijl01@umcg.nl

Supplementary Table S2. Primer sequences

Name	Sequence (5'-3')
**P1**	cgcatgaattccgactcagtcctttcatatacaatatgaagtg
**P2**	atgcgcctgcagcatgttcactccgtttctctatt
**P3**	cgcatgaattcatggataactatcgtgatgaaaaca
**P4**	cgcatggtaccttacgaagttttctcttctttttgatc
**P5**	gttacctggattaattgctgcgtctgtctgaa
**P6**	gcagcaattaatccaggtaaccgtggcggtcctttgttaaatacagacg

Supplementary Table S3. Primer sequences for generation of the Northern Blot probes

Name	Sequence
**amyQ_NOR_for**	gga cag ttt cgt tca gac ttg
**amyQ_NOR_revT7**	gaa att aat acg act cac tat agg gag agc atc agc acc agc ctt atg
**htrA_NOR_for**	atg gat aac tat cgt gat ga
**htrA_NOR_revT7**	gaa att aat acg act cac tat agg gag atg taa tac cga caa tcg ctg

Supplementary Table S4. Reversed phase liquid chromatography (RPLC)

reversed phase liquid chromatography (RPLC)	
instrument	Ultimate 3000 RSLC (Thermo Scientific)
trap column	75 µm inner diameter, packed with 3 µm C18 particles (Acclaim PepMap100, Thermo Scientific)
analytical column	Accucore 150-C18, (Thermo Fisher Scientific)<br>25 cm x 75 µm, 2,6 µm C18 particles, 150 Å pore size
buffer system	binary buffer system consisting of 0.1% acetic acid in HPLC-grade water (buffer A) and 100% ACN in 0.1% acetic acid (buffer B)
flow rate	300 nl/min
gradient	linear gradient of buffer B from 5% up to 25% in 120 min<br>0 min: 2%B<br>2min 5% B<br>10min 5% B<br>130 min: 25%B<br>135 min 40 %B<br>137 min 90 %B<br>142 min 90 %B<br>145 min 2 %B<br>150 min 2 %B
column oven temperature	40°C

Supplementary Table S5. Instrument setup and methods for data independent acquisition mode

Mass spectrometric analysis	
instrument	Q Exactive HF
electrospray	Nanospray Flex Ion Source
operation mode	data-independent (DIA)
Full MS	
Polarity	positive
MS scan resolution	60000
AGC target	5e6
maximum ion injection time for the MS scan	200 ms
Scan range	333 to 1650 m/z
Spectra data type	profile
dd-MS2	
Polarity	positive
Resolution	30,000
MS/MS AGC target	3e6
maximum ion injection time for the MS/MS scans	auto
Spectra data type	profile
selection for MS/MS	1
isolation window	56 windows, 13 m/z
Fixed first mass	-
dissociation mode	higher energy collisional dissociation (HCD)
normalized collision energy	27.5%

Supplementary Table S6. Settings for spectronaut analysis

Spectronaut^{TM} parameters used for analyses Data analyses DIA	
software	Spectronaut version 16 (BiognoSYS, Schlieren, Switzerland)
Pulsar Search	
Peptides	
Enzyme/Cleavages Rules	Trypsin/P
Digest Type	specific
Max Peptide Length	52
Min Peptide Length	7
Missed Cleavages	2
Toggle N-terminal M	True
Labelling	false
Modifications	
Max variable Modifications	5
Fixed Modifications	0
Variable Modifications	Oxidation (M)
Identification	
PSM FDR	0.01
Peptide FDR	0.01
Protein Group FDR	0.01
directDIA Workflow	directDIA+(Deep)
PTM location filter	false
Tolerances	
Calibration Search	Dynamic, MS1 and MS2 Correction Factor 1
Main Search	Dynamic, MS1 and MS2 Correction Factor 1
Workflow	
Fragment Ion Selection Strategy	Intensity Based
In silico generate Missing Channels	False
Results Filters	
Fragment Ions	
Ion AA length	3

Ion Charge /Ion Loss Type/Ion Type	false
m/z	min 300 max 1800
Relative intensity	min 5
Precursors	
Amino Acids	false
Best N Fragments per Peptides	max 10, min 6
DIA Analysis	
XIC extraction	
XIC RT extraction window	dynamic, correction factor 1
MS1 Mass Tolerance Strategy	dynamic, correction factor 1
MS2 Mass Tolerance Strategy	dynamic, correction factor 1
Calibration	
MZ Extraction Strategy	Maximum intensity
Precision iRT	true
Exclude De-amidated Peptides	true
iRT <-> RT Regression Type	Local (Non-Linear) Regression
MS1 Mass Tolerance Strategy	System Default
MS2 Mass Tolerance Strategy	System Default
Identification	
Precursor Qvalue Cufoff	0.001
Precursor PEP Cutoff	0.2
Protein Qvalue Cutoff (Experiment)	0.01
Protein Qvalue Cufoff (Run)	0.05
Protein PEP Cufoff	0.75
Single Hit Definition	By stripped sequence
Pvalue Estimator	Kernel Density Estimator
Quantification	

Precursor Filtering	Identified (Qvalue)
Imputation Strategy	Use background signal
Proteotypicity Filter	None
Protein LFQ Method	Automatic
Quantity MS level	MS2
Cross run normalization	Local normalization, row selection: Identified in at least one run (sparse)
Interference Correction	Only identified peptides,<br>exclude all multi-channel interferences MS1 min 2, MS2 min3
Major (Protein) Grouping	By Protein Group ID
Minor (Peptide) Grouping	By Stripped Sequence
Major Group Quantity	Mean peptide quantity
Major Group Top N	max 3 min 2
Minor Group Quantity	Sum precursor quantity
Workflow	
Profiling strategy	iRT profiling
Carry-over exact Peak Boundaries	false
Profiling Row Selection	Minimum Qvalue row selection, Qvalue treshold 0.001
Profiling Target Selection	Profile only non-identified precursor,<br>Identification Criterion: Qvalue, treshold 0.001
Run Limit for directDIA Library	-1 (use all DIA files for library generation)
Unify peptide peaks	Select corresponding peak
Protein Interference	
Protein Interference Workflow	automatic
Interference Algorithm	IDPicker
